# Visual MODFLOW, solute transport modeling, and remote sensing techniques for adapting aquifer potentiality under reclamation and climate change impacts in coastal aquifer

**DOI:** 10.1038/s41598-024-72933-8

**Published:** 2024-10-01

**Authors:** Moaz M. Abd El Ghany, Shaimaa M. El-Hadidy, Sameh A. Sakr, Ezzat A. Korany, Samah M. Morsy

**Affiliations:** 1 Geology Department, Faculty of Science, Ain Shams University, Cairo, 11566 Egypt; 2 National Water Research Center, Ministry of Irrigation and Water Resources (MWRI), Giza, Egypt

**Keywords:** Groundwater development, Visual MODFLOW, Solute Transport (MT3DMS), Climate change, Global warming, Environmental sciences, Hydrology

## Abstract

Global environmental changes, such as climate change and reclamation alterations, significantly influence hydrological processes, leading to hydrologic nonstationarity and challenges in managing water availability and distribution. This study introduces a conceptual underpinning for the rational development and sustainability of groundwater resources. As one of the areas intended for the development projects within the Egyptian national plan for the reclamation of one and a half million acres; hundreds of pumping wells were constructed in the Moghra area to fulfill the reclamation demand. This study investigates the long-term impacts of exploiting the drilled pumping wells under climate change. The approach is to monitor the groundwater levels and the salinity values in the Moghra aquifer with various operational strategies and present proposed sustainable development scenarios. The impact of global warming and climate change is estimated for a prediction period of 30 years by using satellite data, time series geographical analysis, and statistical modeling. Using MODFLOW and Solute Transport (MT3DMS) modules of Visual MODFLOW USGS 2005 software, a three-dimensional (3D) finite-difference model is created to simulate groundwater flow and salinity distribution in the Moghra aquifer with the input of forecast downscaling (2020–2050) of main climatic parameters (PPT, ET, and Temp). The optimal adaptation-integrated scenario to cope with long-term groundwater withdrawal and climate change impacts is achieved when the Ministry of Irrigation and Water Resources (MWRI) recommends that the maximum drawdown shouldn’t be more significant than 1.0 m/ year. In this scenario, 1,500 pumping wells are distributed with an equal space of 500 m, a pumping rate of 1,200 m^3^/day and input the forecast of the most significant climatic parameters after 30 years. The output results of this scenario revealed a drawdown level of 42 m and a groundwater salinity value of 16,000 mg/l. Climate change has an evident impact on groundwater quantity and quality, particularly in the unconfined coastal aquifer, which is vulnerable to saltwater intrusion and pollution of drinking water resources. The relationship between climate change and the hydrologic cycle is crucial for predicting future water availability and addressing water-related issues.

**Supplementary Information:**

The online version contains supplementary material available at 10.1038/s41598-024-72933-8.

## Introduction

Water resource availability is an essential requirement for all socioeconomic development projects. Given the growing significance of water resources management to fostering sustainable development in the Mediterranean region, it is imperative to protect aquifer potentiality and water quality. The construction of the Grand Ethiopian Renaissance Dam (GERD) will decrease the water discharge in the Nile River by about 11 and 19 billion m^3^ (BCM)^1^. Additionally, the freshwater-saltwater interface is dynamically dependent on the change in the hydraulic head^2^ and the natural gradient of freshwater is reduced with increasing pumping, Therefore, uncontrolled pumping; consecutively leads to dramatic drawdown and increasing groundwater salinization in the Moghra aquifer. Groundwater in coastal and semiarid regions is crucial for drinking water and ecosystems. Uncontrolled aquifer exploitation leads to drawdown, sea level rise, and increased groundwater salinization^2^.

Groundwater models are mathematical representations of groundwater systems, including assumptions for different scenarios. Models are applied to describe the hydrogeological processes of flow, solute transport, and transformation. Groundwater modeling is essential in hydrologic studies, allowing for the simulation of water movement through aquifers. It helps evaluate groundwater quantity and quality and allows for scenario construction based on factors like abstraction rates, human activities, and environmental conditions. Evaluating models requires defining study objectives, understanding their strengths and limitations, and comparing conceptual models to computer model programs. Groundwater modelling is crucial for managing groundwater systems, providing a theoretical framework for understanding dynamics and human intervention. It aids in water resource assessment, conservation, and restoration, offering cost-effective insights for new strategies and legislation.

The global population growth and rising water demand have led to a significant reduction in groundwater storage, with studies showing links between climate variations and groundwater levels. This demand, particularly in rural and desert regions, is expected to significantly shape water resource management and food security, with direct impacts from temperature, precipitation, and CO^2^ concentrations. Many researchers studied climatic change and groundwater levels and water availability^3^^,^^4^^,^^5^^,^^6^ and^7^ analyzed the influence of climate change on groundwater storage, including groundwater level fluctuations, prediction, and management.

Changes in precipitation patterns can lead to more intense and less predictable rainfall events, affecting groundwater recharge rates and causing severe droughts and floods. More frequent, heavy, and intense rainfall events are predicted, but excess runoff can lead to water pollution and limited access for humans and ecosystems^3^. Rising temperatures increase evaporation rates and water demand, reducing surface water availability and stressing aquifers, especially unconfined ones. Sea-level rise can lead to saltwater intrusion into coastal aquifers, contaminating freshwater resources and reducing usable groundwater availability^7^. Extreme weather events, such as droughts and floods, can directly impact groundwater levels, decreasing recharge and potentially causing contamination from surface pollutants.

Climate change has a direct influence on groundwater systems, including recharge, discharge, and temperature. Hydrological models are crucial in comprehending the impact of climate change on groundwater and guiding management decisions. Advanced models integrate data assimilation and enhanced process representation, which help to comprehend complex systems. Advances in hydrological modeling, such as uncertainty quantification and remote sensing data, may enhance models for analyzing climate change effects. Impact on groundwater remains uncertain due to its intricate nature^8^. The Intergovernmental Panel on Climate Change predicts a 0.6 ± 0.2 ◦C increase in global mean surface temperature since 1861, affecting hydrological processes, precipitation patterns, timing, and intensity. Researchers have utilized hydrological models to assess the impact of climate change on surface and groundwater resources, specifically focusing on groundwater recharge, which is influenced by hydrological processes. This study explores recent methodologies, tools, techniques, and spatial/temporal assumptions in groundwater vulnerability assessment, emphasizing the importance of indicator choice for hybridization. The decision for model hybridization is shown to demonstrate the research need. Amanambu et al. 2020^9^, study climate change impacts groundwater interactions, flows, recharge, storage, discharge, and quality. Land use changes, vegetation and cultivation techniques, and increased crop evapotranspiration water demand pressure groundwater availability, affecting its recharge and storage. The water availability has three interconnected components: precipitation, temperature, and ET. Changes in one parameter can have a complicated effect on the order of the stream and the direction of the runoff. For example, rising temperatures can cause increased ET, lowering soil moisture and potentially modifying precipitation patterns, all which impact runoff and stream formation.

The satellite monitoring technology is crucial in sustainable management. Extreme weather events, such as droughts and floods, can directly impact groundwater levels, decreasing recharge and potentially causing contamination from surface pollutants^6^.

The evaporation rate determines the amount of run-off water, primarily by solar radiation, temperature, relative humidity, and wind speed. Temperatures and droughts are continuously rising. Temperatures may render certain locations unsustainable in the future, resulting in a decrease in freshwater supply. Groundwater availability and dependence may be negatively impacted by increased precipitation variability and more severe weather events brought on by climate change^5^. Aquifer depletion is more likely during prolonged droughts, especially in shallow unconfined aquifers. Both the amount and the quality of groundwater are impacted by climate change. The rise in sea level may cause coastal aquifers to become contaminated by salt water, compromising groundwater quality. It is important to understand these systems to implement successful management strategies in the face of climate change. Controlled Aquifer Recharge is used to recharge depleted aquifers’ groundwater levels, store water for later use, or prevent saltwater intrusion 10].

In water resources management, it is essential to consider the challenge of climatic change’s impact on the different hydrologic processes. Climate change is reducing water availability, affecting evaporation rates and land degradation. Rising temperatures and droughts may lead to a decrease in freshwater supply. Groundwater availability and dependence are negatively impacted by precipitation variability and severe weather events. Aquifer depletion is more likely during prolonged droughts. Rising sea levels may also compromise groundwater quality^6^. It is important to understand these systems to implement successful management strategies in the face of climate change. Controlled Aquifer Recharge is used to recharge depleted aquifers’ groundwater levels, store water for later use, or prevent saltwater intrusion^10^.

The main objective of this study is to achieve extreme exploitation of the existing pumping wells in the Moghra aquifer with mitigation from deterioration of groundwater quality and adaptation of the influence of global warming and climate change. MODFLOW 2005 USGS groundwater modeling software^11,^^12^ is applied to determine the safe withdrawal of the pumping wells by suggesting alternative pumping scenarios. The solute Transport (MT3DMS) module is applied to simulate the impact of groundwater withdrawal on aquifer salinity. Mathematical models describe and simulate the flow and contaminant transport through a set of equations^13^. Morsy (2023)^14^ applied the visual MODFLOW model to evaluate the effect of various operational scenarios on the groundwater aquifer. El-Hadidy and Morsy^15^ integrated the groundwater visual MODFLOW model with remote sensing and GIS techniques to detect the expected changes in the groundwater system and land cover due to reclamation. Gomma et al. (2021)^16^ used the SEAWAT module to assess how much the pumping wells will draw seawater to the Moghra aquifer system.

## Description of the study area

**Fig. 1:**
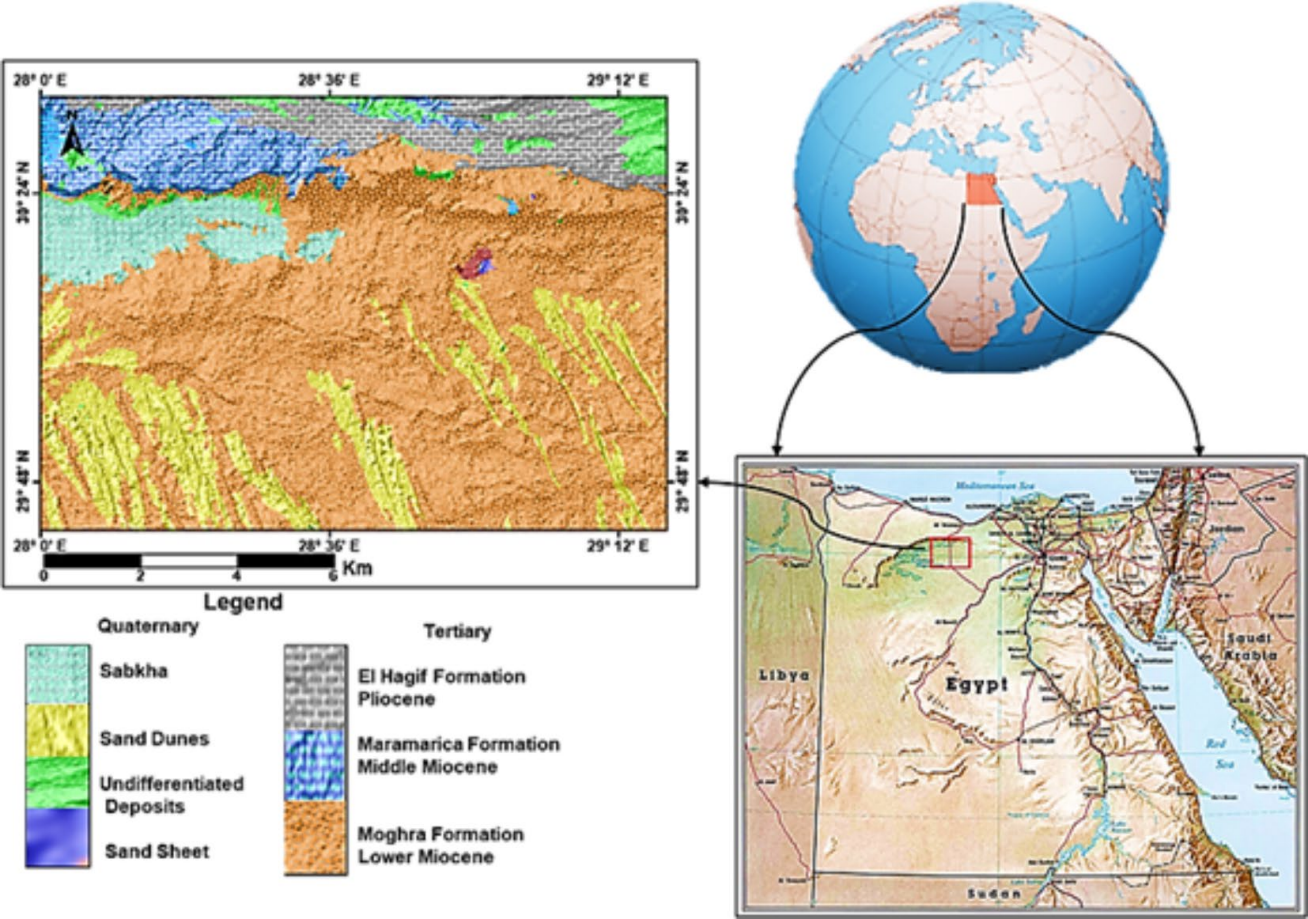
Location map of the study area and geological map modified after COCNO (1987)^27^.

**Fig. 2:**
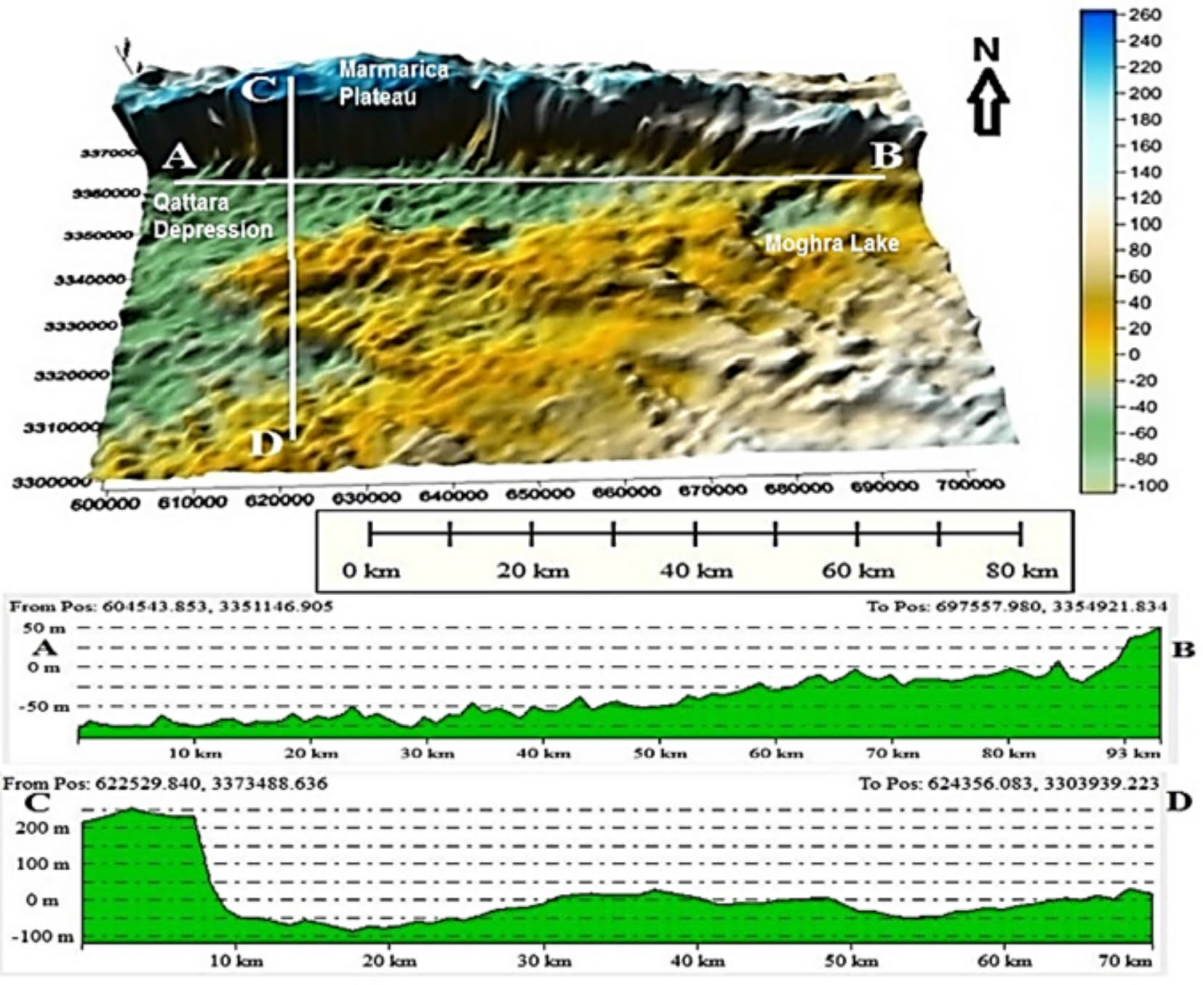
Geomorphologic units and Digital Elevation Model (DEM) of the Moghra area, Western Desert, Egypt.

The Moghra area occupies the northern portion of the Western Desert to the east of the Qattara Depression (Fig. 1). It belongs to arid to semiarid regions with temperature ranges from 36.2 °C during summer months to 6.2 °C during winter months^17^. In the northern part, the annual rainfall ranges between 25 and 50 mm, while in the southern part, it reaches 25 mm^18^. The average evaporation rate is 330 mm/year^19^. The humidity varies between 39.5 and 19% from December to June^20^. Significant variations in ground elevation appeared in various geomorphic units (Fig. 2). Marmarica plateau extending in the North with topographic elevation reaches 250 m (m.s.l.) and decreases sharply forming a cliff at its southern part. The Qattara Depression has the lowest topographic elevation of -100 (m.s.l.) on the western side, which is covered by sabkhas and salt marshes. Along the southeastern side of the Qattara Depression, there is a series of elongated sand-dunes chains ranging in elevation between 20 and 100 m (m.s.l.).

The study area is covered by geologic rock units ranging in age from the lower Miocene to the Quaternary, constituting lithostratigraphic units (Fig. 1). The Lower Miocene Moghra formation is differentiated into three members El Raml, Bait Owain, and Monquar El Dowi members^21^. These three members are regarded as one hydrogeologic unit as they are hydraulically connected^22,23^. Possible recharge sources to the Moghra aquifer are rainfall precipitation, Mediterranean seawater^24,25^, groundwater from the Nubian Sandstone aquifer system, and the Quaternary aquifer in the western Nile Delta^26^. In Egypt, the Nile River water did not satisfy the cities’ growing demands, so the Egyptian government decided to reclaim 1.5 million acres in different regions in the Western Desert of Egypt. The Moghra area is one of these scoped regions. The Moghra aquifer as an unconfined shallow aquifer is vulnerable to climate change, especially sea-level rise, the low terrain in the Moghra area besides its proximity to the sea level rendered the aquifer at risk from a high salinity concentration.

## Methodology

**Fig. 3:**
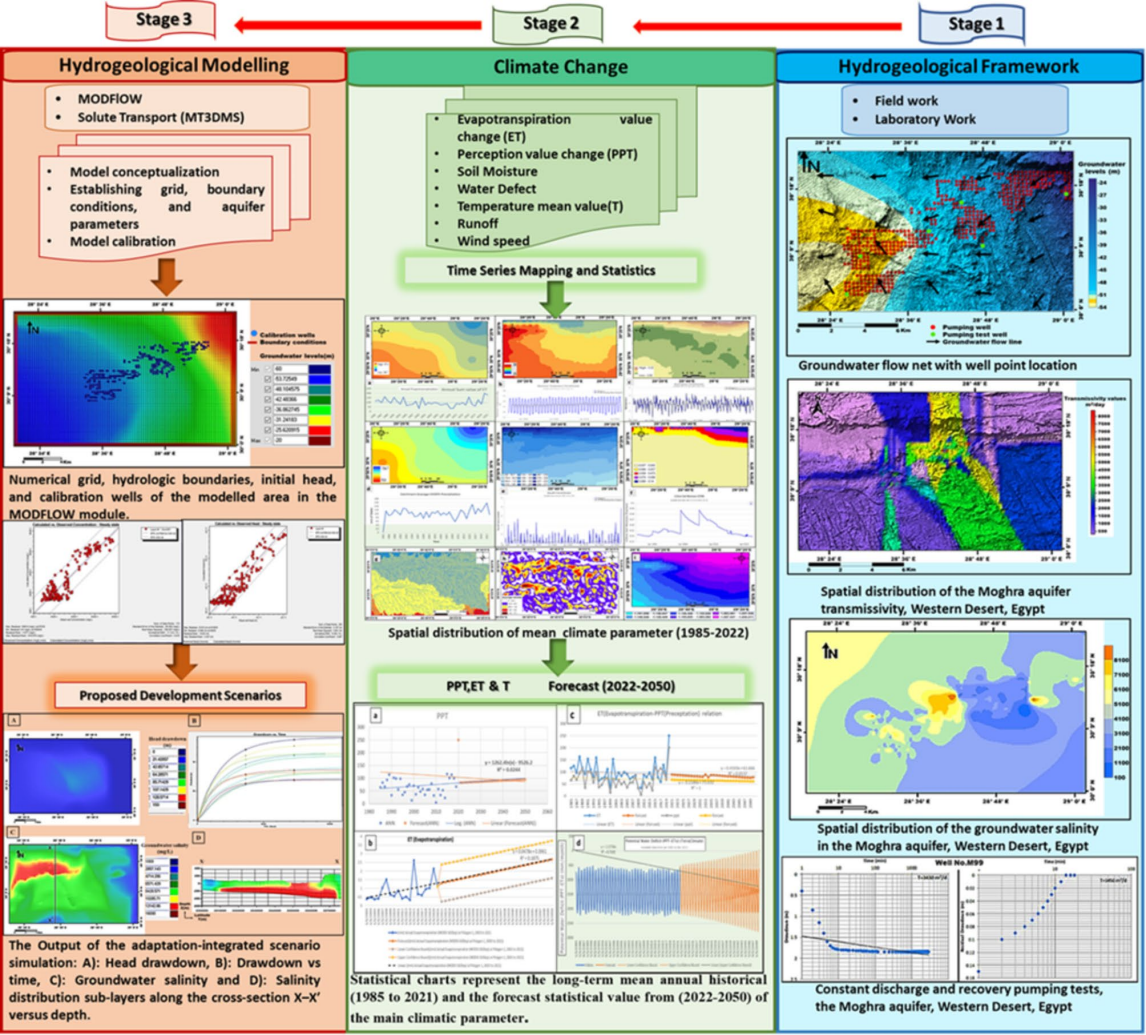
Stages for work procedure of the present study.

The work procedure for this study took place through three main stages, which can be summarized in Fig. 3. The study uses satellite data analysis to identify a decreasing precipitation trend in the Moghra aquifer region and an increase in surface temperature. The study reveals a strong positive statistical correlation between reduced precipitation and declining groundwater levels, with regression models indicating that temperature increases significantly influence variations in groundwater recharge rates. Satellite data can be integrated with ground-based observations, climate time series models, and hydrological models to enhance understanding and management strategies. MODFLOW simulations using climatic data to predict future groundwater levels in the Moghra aquifer, revealing significant climatic influences on groundwater fluctuation and potency variations. The analysis process involves integrating satellite-derived climatic data with ground-based observations of groundwater levels, using GIS for data management, conducting sensitivity analysis to understand climatic variables’ impact on groundwater levels, simulating different scenarios to predict future fluctuations, finding a strong positive correlation between reduced precipitation and declining groundwater levels, and running MODFLOW simulations to predict future groundwater levels under different climate scenarios.

**Fig. 4:**
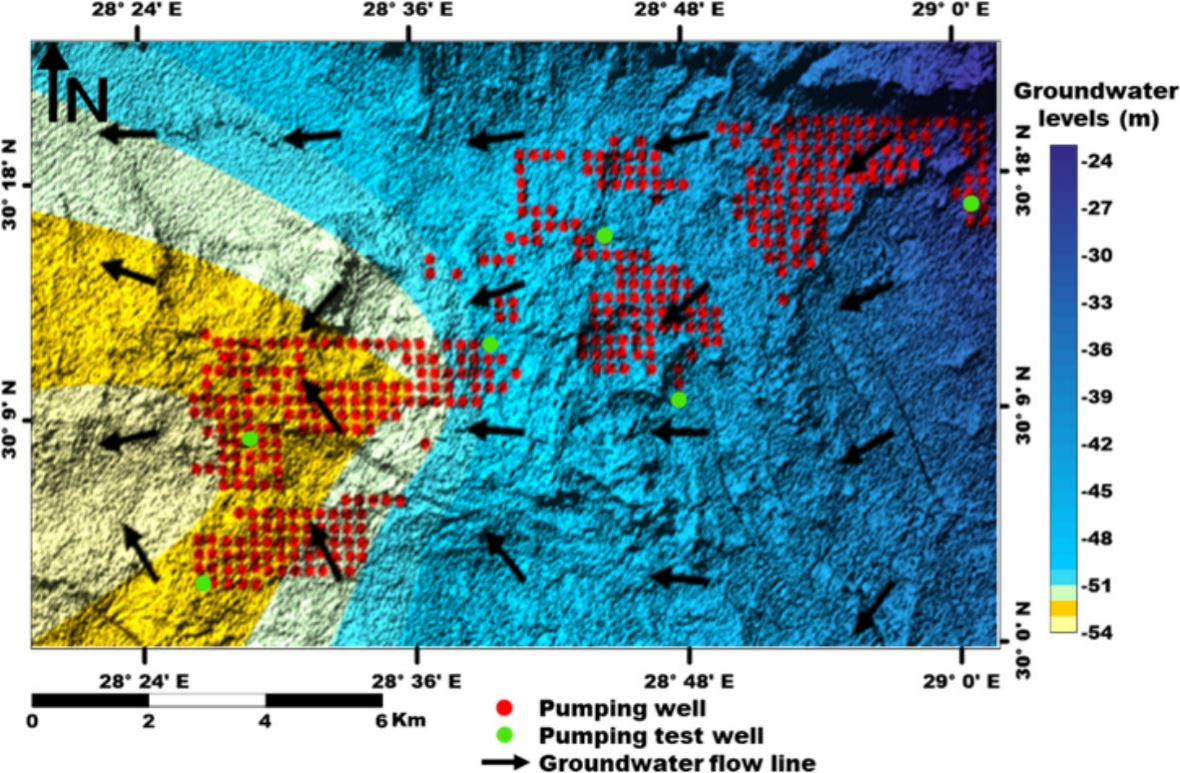
Groundwater flow net of the Moghra aquifer, and sample sites, Western Desert, Egypt.

Fieldwork and collecting data of about 400 production wells^28^, their locations are shown in Fig. 4 are carried out to fulfil the aim of this study. The implemented technique is the Visual MODFLOW model and links the forecasted climatic parameters to obtain potential future groundwater extraction scenarios in the shallow Moghra aquifer under climate change and reclamation impact.

### Hydrogeological framework

**Fig. 5:**
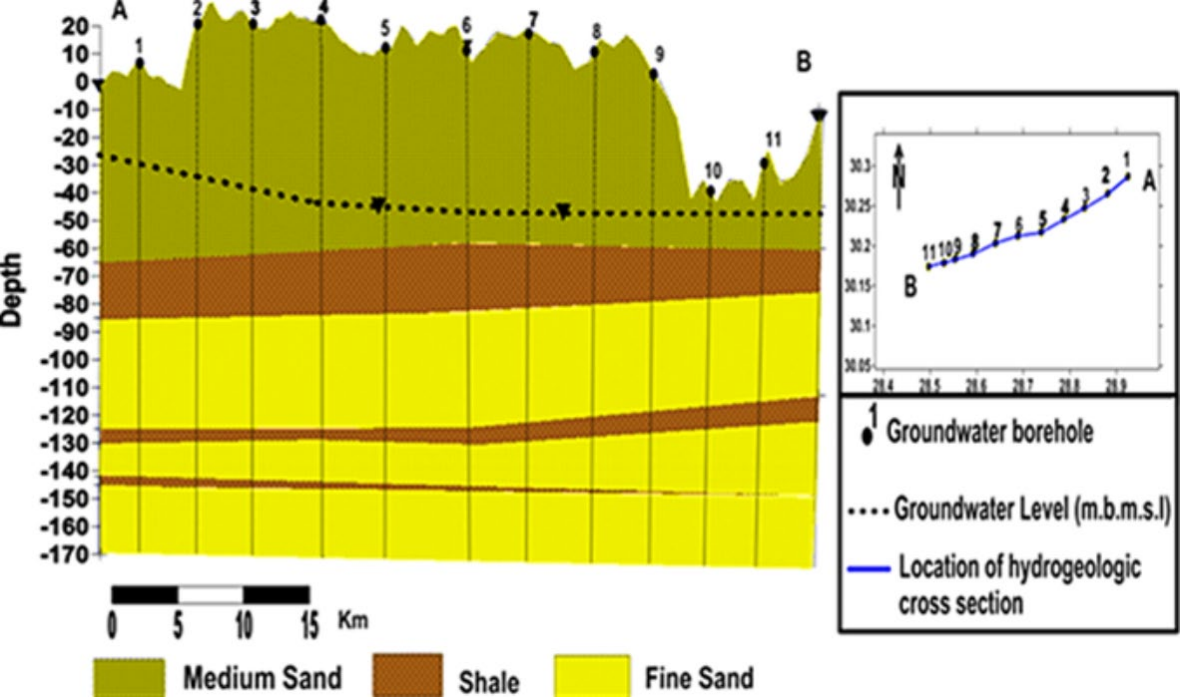
Hydro-stratigraphic cross-section, the Moghra aquifer, Western Desert, Egypt.

**Fig. 6:**
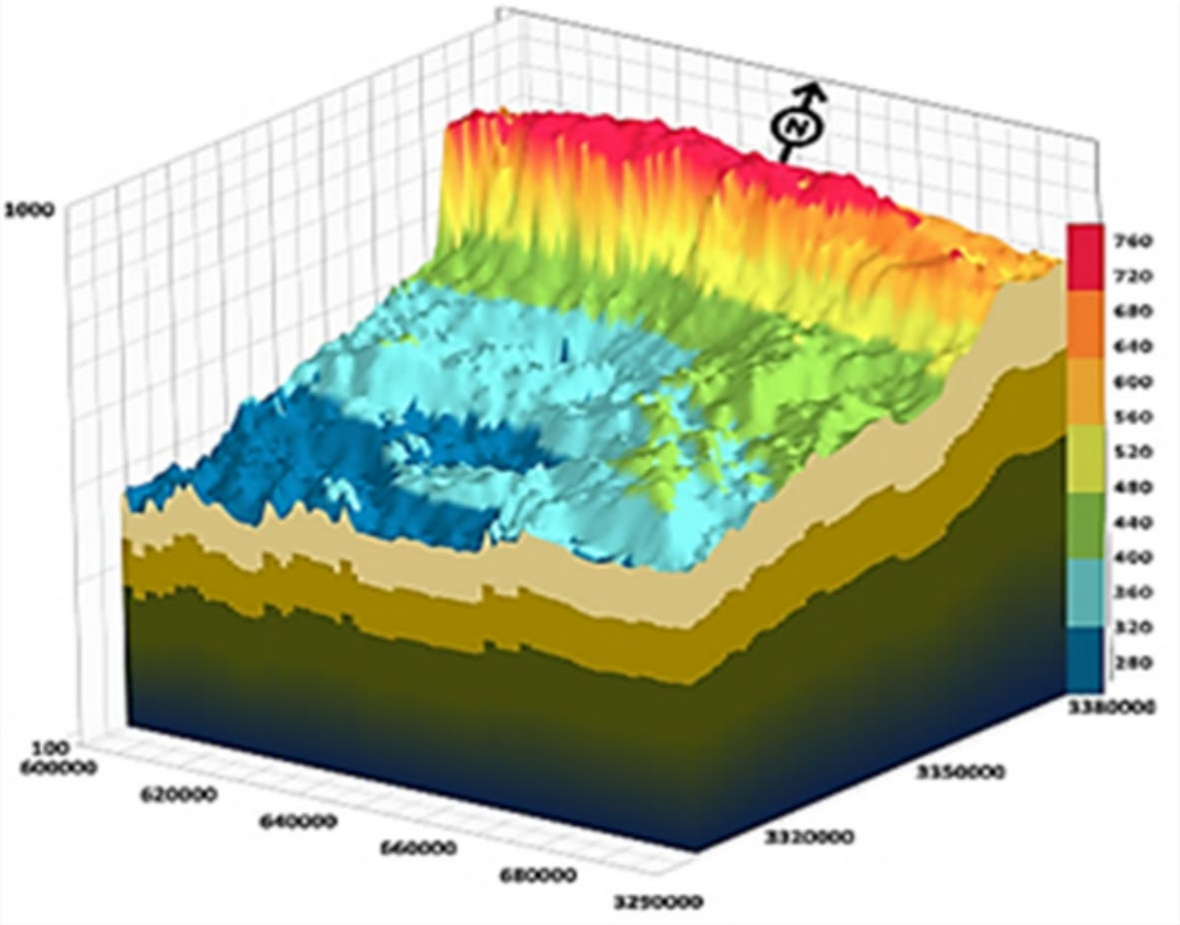
Block diagram shows the Moghra aquifer thickness, Western Desert, Egypt.

**Fig. 7:**
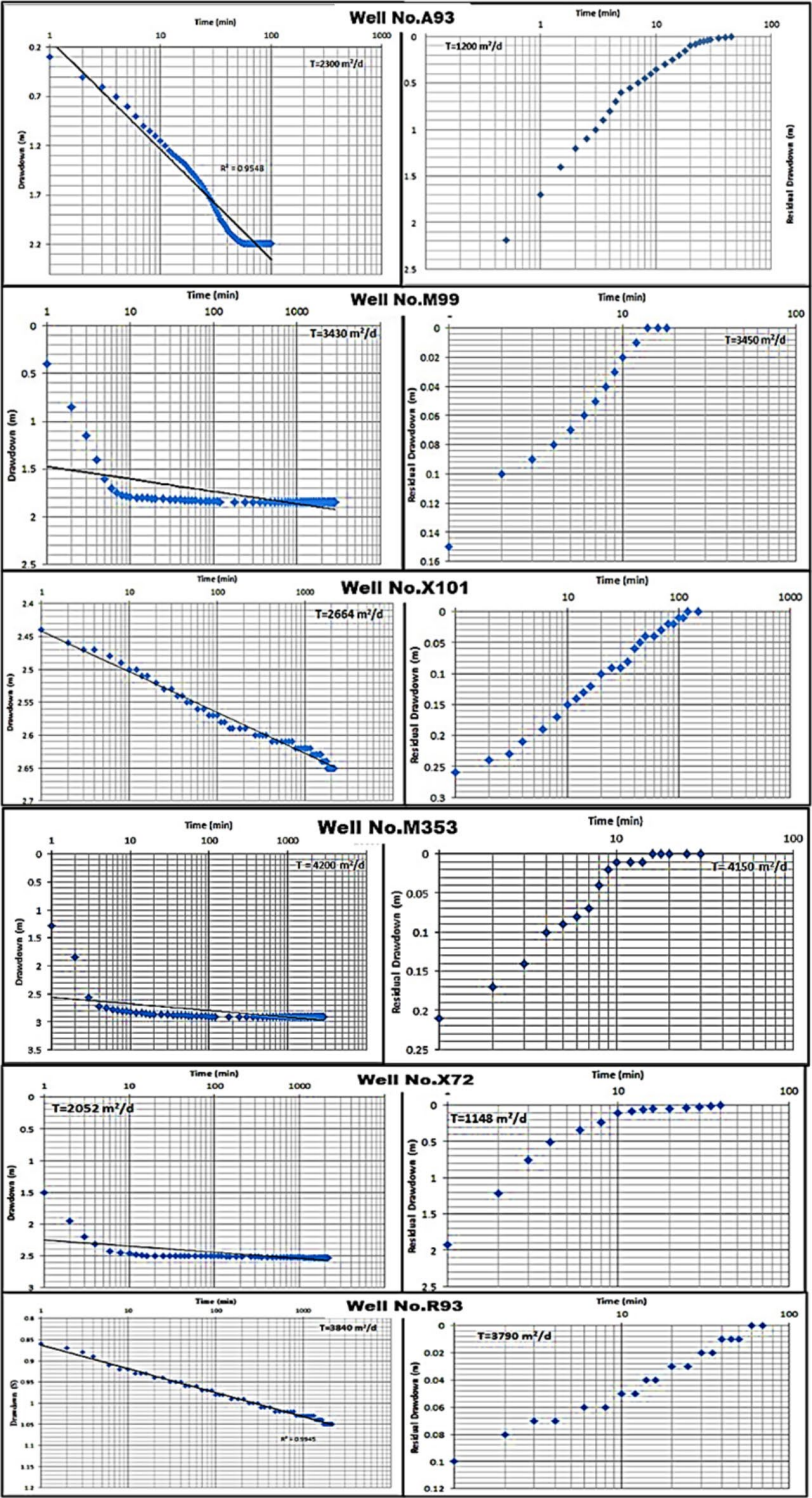
Constant discharge and recovery pumping tests, the Moghra aquifer, Western Desert, Egypt.

**Fig. 8:**
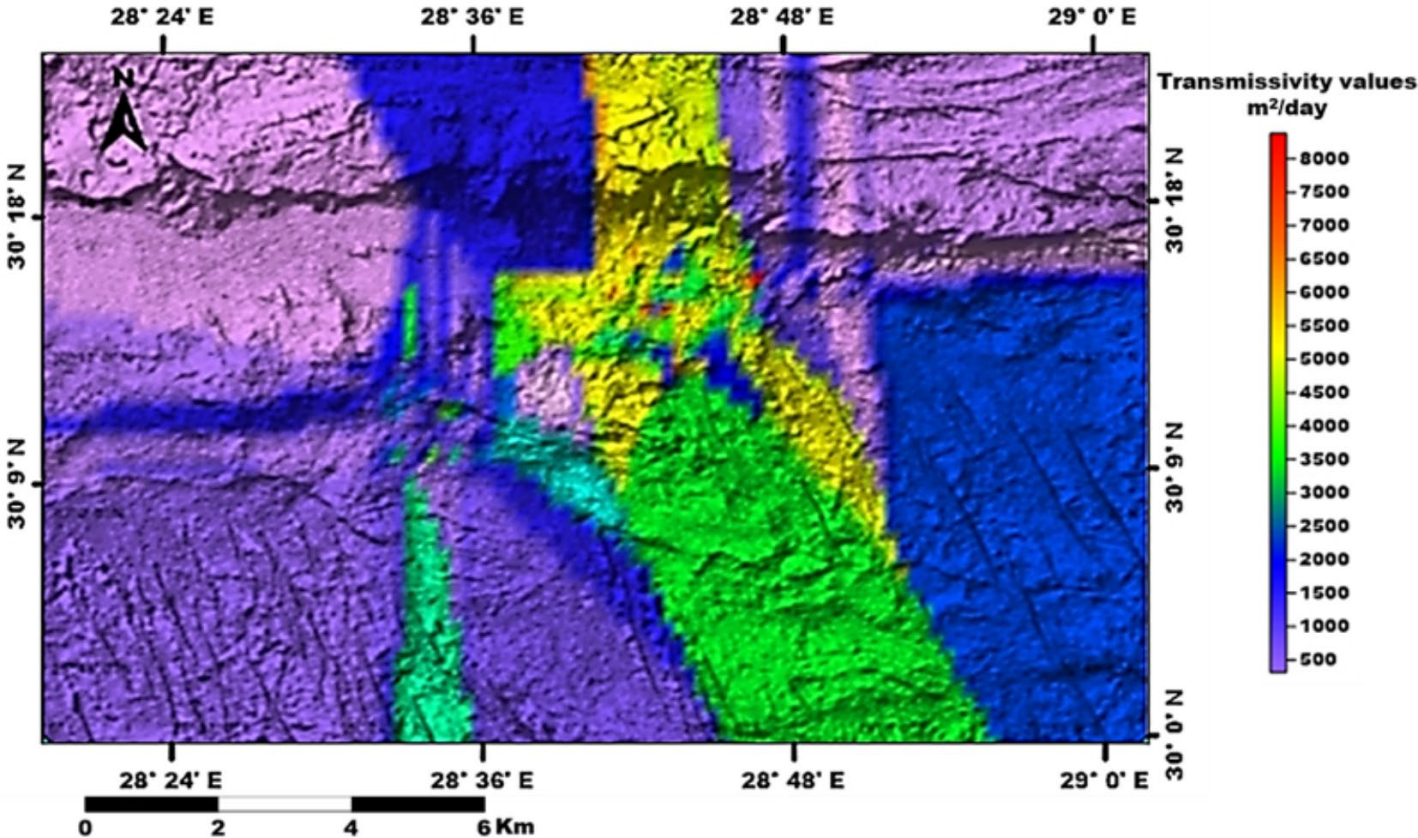
Spatial distribution of the Moghra aquifer transmissivity, Western Desert, Egypt.

**Fig. 9:**
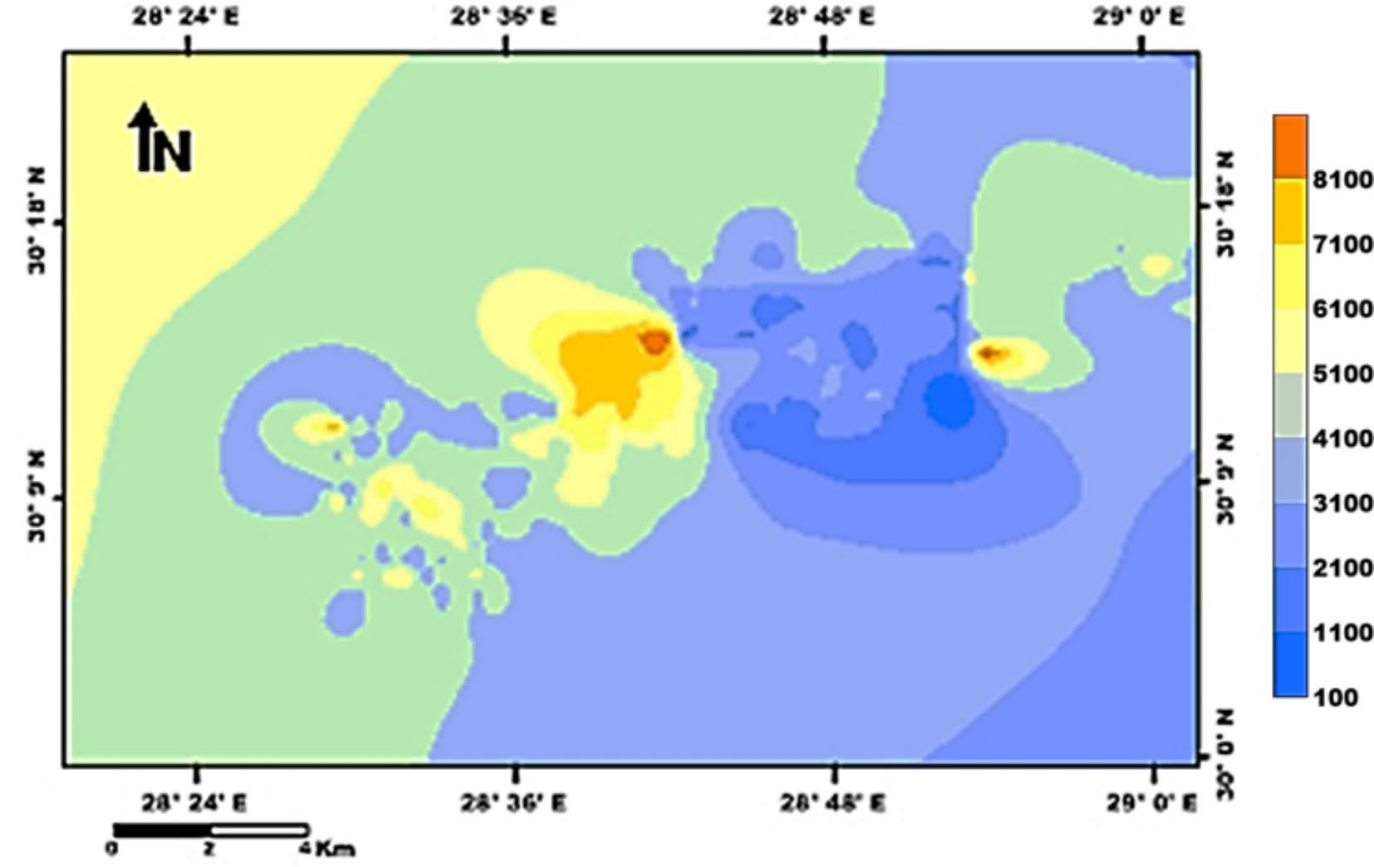
Spatial distribution of the groundwater salinity in the Moghra aquifer, Western Desert, Egypt.

The groundwater in the Moghra aquifer in the study area occurs under unconfined conditions, generally flowing from the east and northeast to the west direction. The groundwater levels range from − 20 m (m.s.l) in the eastern part of the study area to -55.78 m (m.s.l.) close to the Qattara Depression forming discharge area (Fig. 4). Figure 5 shows the hydro-stratigraphic cross-section in the study aquifer. The aquifer thickness increases due to the north reaching 500 m (Fig. 6). To estimate aquifer hydraulic parameters, pumping test analyses are conducted on the existing pumping wells applying Jacob’s straight-line method^29^ and Theis’s recovery methods^30^. Figure 7 shows representative constructed curves of pumping test analyses for selected wells’ locations are shown in Fig. 4. The resulting aquifer transmissivity values range from 497 to 9,390 m^2^/ day (Fig. 8). The spatial distribution map of groundwater salinity in the Moghra aquifer is represented in (Fig. 9); it reflects a recharge source of fresh water from the east and southeast directions and an increase in salinity due to the west (discharge area) and the north (towards the sea) almost coinciding with the groundwater flow direction.

### Historical meteorological time series data analysis and mapping

**Fig. 10:**
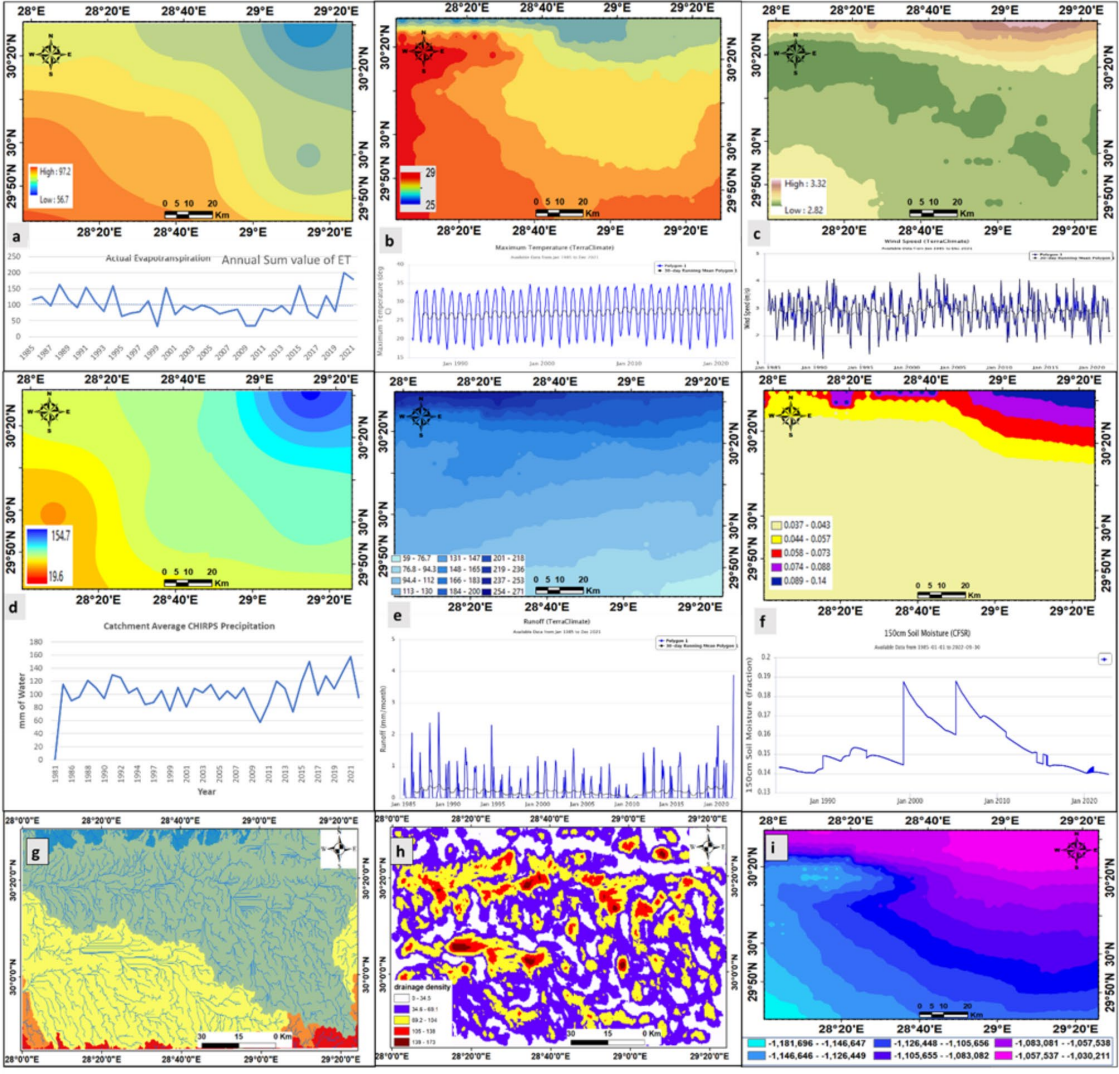
Spatial distribution of mean annual historical (1985 to 2022) mapping and its representative charts, (**a**) actual potential evapotranspiration (APET), (**b**) max-temperature, (**c**) wind speed, (**d**) rainfall precipitation, (**e**) run-off, (**f**) 150 cm soil moisture, (**g**) drainage basin, (**h**) drainage density, and (**i**) water deficit (PPT rainfall less areal potential evapotranspiration) across the study area.

As determined by satellite data, time series geographical analysis, and statistical modelling, groundwater fluctuation and potency variations in the unconfined Moghra aquifer are significantly influenced by climatic conditions. Elevation and slope data were acquired from the digital elevation model (SRTM 1-ArcSecond Global-DEM) using USGS satellite data with a spatial resolution of 30 (http://earthexplorer.usgs.gov/) with WGS_1984_UTM_Zone_36N projection in ArcGIS 10.3 software. Historical and current daily and monthly climate grids for the parameters - such as precipitation, temperature, actual evapotranspiration, runoff, wind speed and recharge, and surface runoff of the studied region utilized to build the drainage pattern and stream order (Fig. 10) and to identify the direction of flow of the runoff water. Long-term climate and streamflow data are examined to determine trends, correlations, and causal links between climatic factors that influence soil moisture, infiltration rates, streamflow dynamics, and hydrological reactions. To anticipate streamflow and runoff patterns, the Hydrologic Modelling System simulates hydrological processes within a watershed, considering precipitation, temperature, ET, and other variables. Precipitation, temperature, and actual evapotranspiration (AET) are all crucial factors in hydrology. High precipitation can increase stream flow, while intense rainfall can cause flash floods. Temperature affects hydrological processes, while AET reduces available water for streamflow. The stream order categorizes streams based on their tributary network, and runoff direction is influenced by topography. High precipitation and steep slopes can lead to faster runoff.

Meteorological time series and weather station data from 1985 to 2022 were used to analyze climate variability in the GIS environment. Climate Hazards Group InfraRed Precipitation with Station data (CHIRPS): Rainfall Estimates from Rain Gauges and Satellite Observations like gridded satellite-based precipitation estimates from NASA and NOAA have been leveraged to build high-resolution (0.05°) gridded precipitation climatologist, https://data.chc.ucsb.edu/products/CHIRPS-2.0. Long-term time series data set from 1985 to 2022 of Temperature, evapotranspiration, windspeed, and run-off time series data obtained from the Terraclimate dataset of climate, climatic water balance for global terrestrial surfaces, with high-spatial resolution 4 km. The yearly aggregates of the variables were utilized in all situations. Downloaded from WorldClim and climate engine website. long-term potential evapotranspiration (PET) data with Moderate Resolution Imaging 500 m, Spectroradiometer (MODIS) remotely sensed data products (MOD16 global evapotranspiration product), the dataset covers the time 2000 through 2010, with a 16-bit unsigned integer downloaded from NASA Earthdata (LP DAAC): MOD16A2 Data. Long-term time series data set from 1985 to 2022 of Temperature, evapotranspiration, windspeed, and run-off time series data obtained from the Terraclimate dataset of climate, climatic water balance for global terrestrial surfaces, with high-spatial resolution 4 km. The yearly aggregates of the variables were utilized in all situations. Downloaded from WorldClim and climate engine website. long-term potential evapotranspiration (PET) data with Moderate Resolution Imaging 500 m, Spectroradiometer (MODIS) remotely sensed data products (MOD16 global evapotranspiration product), the dataset covers the time 2000 through 2010, with a 16-bit unsigned integer. Three global warming scenarios (GCMs) to assess changes in groundwater recharge rates in 2050.

The climate change scenarios used in this study CMIP5 climate models have been used in modelling studies. In the business-as-usual scenario, the likely climate-driven consequences of GWS changes over the twenty-first century were examined (RCP8.5)^31^. Soil moisture data can be collected by satellites measuring microwaves reflected or emitted by Earth’s surface. The intensity of the measured signal depends on the amount of water in the soil downloaded from https://gimms.gsfc.nasa.gov. The intensity of the measured signal depends on the amount of water in the soil. Since the 1970s, microwave remote sensing techniques have been used to collect surface soil moisture, also known as the water content of the topmost soil layer, at various temporal and geographical scales^32^. The link between surface temperature (LST) and the normalized difference vegetation index (NDVI) is based on soil moisture value experimental characterization ^33^.

The amount of additional water plants could utilize when accessible is referred to as the climatic water deficit. This contributes to our understanding of ecological drought, the water stress experienced by plants, which has a cascade effect on animals and stream flow. The model calculates a water stress factor, comparing actual available water to PET, to scale down to actual ET based on water deficit. Meteorological data like temperature, vapor pressure deficit, and solar radiation are used to estimate PET. High temperatures and low humidity increase PET, while actual ET depends on soil moisture availability. the climatic change water deficit used as an indicator for drawdown in groundwater storage especially the studied Moghra aquifer is unconfined and affected by changes in climatic parameters, Fig. 10.

This contributes to our understanding of ecological drought, the water stress experienced by plants, which has a cascade effect on animals and stream flow. Evaluating and modelling forecasts based on historical Climate time series recorded data. It entails developing models based on past data and employing them to make observations, drive the future, make forecasts, and inform strategic decision-making.

### Model conceptualization

Development of the conceptual model is based on the description of the geologic setting, identification of the hydrogeologic processes, and collection of field data. Mathematical models employ governing equations resulting from the translation of physical laws and applying proper simplifying assumptions including equation(s) that represent necessary phenomena occurring in the conceptual model. The simplest mathematical model of groundwater flow is Darcy’s Law^34^. An example of a more sophisticated groundwater model addresses 3-dimensional transient groundwater flow and solute transport. The present work applies the MODFLOW and Solute Transport (MT3DMS) modules of Visual MODFLOW USGS 2005 software (McDonald and Harbaugh, 1988)^11^ to simulate groundwater flow and salinity distribution in the Moghra aquifer. Visual MODFLOW and ArcGIS are integrated to regulate the database, create spatial analysis, and obtain thematic maps. The model includes grid design, setting boundary, initial conditions and aquifer hydraulic parameters.

1.1. Hydrostratgraphic units.
1.2. Digital Elevation Model by ASTER (Dem) 1-ARC resolution with 30 m is imported as the upper surface of the Moghra aquifer (Fig. 2). The aquifer system is represented in the model by two hydrostratigraphic units, the upper one is the lower Miocene Moghra unconfined aquifer which is formed of three hydraulically connected members, while the Oligocene layer represents the base confining unit.Aquifer hydraulic properties.

**Fig. 11:**
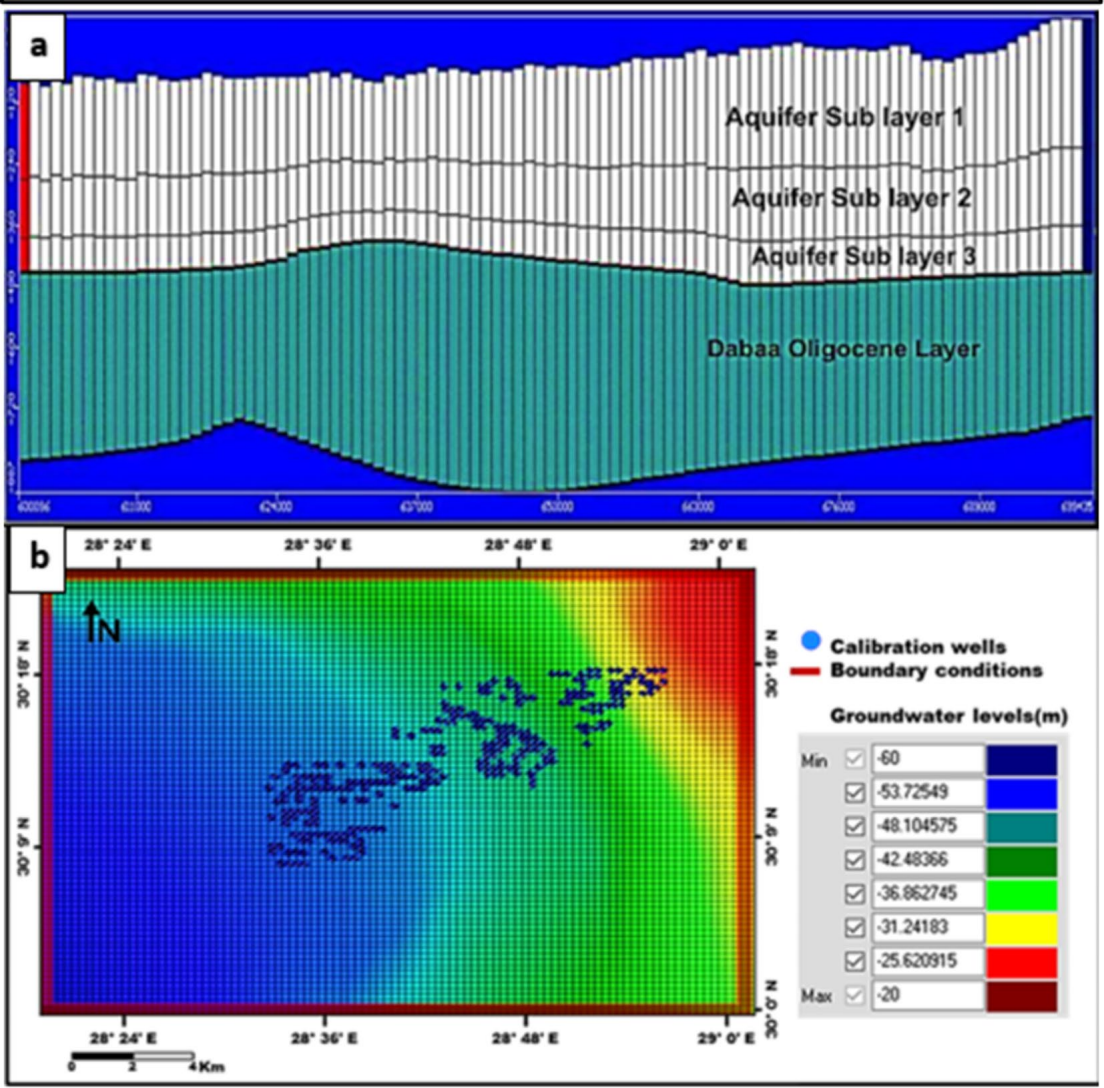
**a** Constructed hydrostratigraphic units, **b** Numerical grid, hydrologic boundaries, initial head, and calibration wells of the modelled area in the MODFLOW module.

The model hydraulic parameters of transmissivity (T), and hydraulic conductivity (k) values are estimated from the results of constant discharge, and recovery tests for the wells at the modelled area (Table 1) (Fig. 7). The current climatic data estimated from Rain Gauges and Satellite Observations are inserted into the model (Fig. 11a).

**Table 1:** Hydraulic parameters of the Moghra aquifer, Western Desert, Egypt.

Well No	Pumping Rate (m^3^/hr)	D.D (m)	Well depth (m)	T (pumping) (m^2^/d)	T (Recovery) (m^2^/d)	Average K (m/d)
PW-A93	100	2.19	167	2300	1200	13.83
PW-M99	100	1.85	124	3430	3450	15.9
PW-M353	100	2.9	115	4200	4150	26.9
PW-R93	100	1.05	124	3840	3790	24.25
PW-X72	100	2.53	126	2052	1148	13.13
PW-X101	100	2.66	126	2664	2590	26.26

1.3. Groundwater flow system.

**Fig. 12:**
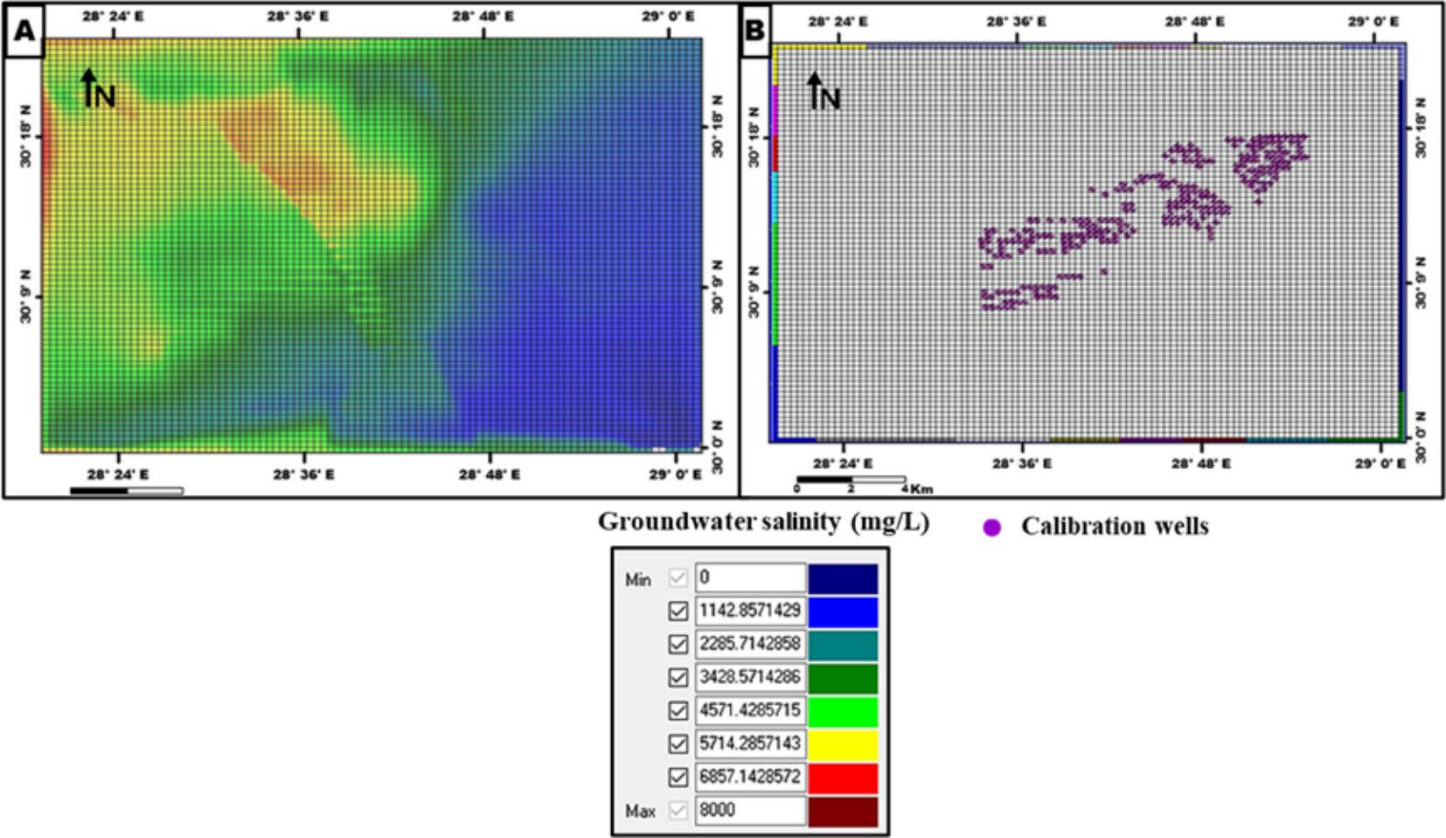
**A** Numerical grid and initial concentration and **B** Concentration boundaries and calibration wells, the modelled area in the MT3D module.

The groundwater flow direction is from E-NE to W-SW. The flow net of the Moghra aquifer (Fig. 4) reflects a recharge area to the east of the study area and a discharge area to the west at the Qattara Depression. The current head distribution in the aquifer is used as an initial condition for the modelled area at the beginning of the simulation. For the Solute Transport Model, constant concentration boundaries are assigned for the modelled area The current salinity distribution is used as the initial concentration for solute simulation (Fig. 12).

1.4. Model boundary conditions.

In a three-dimensional finite-difference grid, the study area is discretized into 78 rows and 100 columns in uniform square cells with 1000 m x 1000 m cell size (Fig. 11b). To represent alternative boundary conditions, equations specify the value of head, flux, or an equation relating a dependent flux to a dependent head. Based on the dominant hydrological conditions of the aquifer; constant head boundaries (Dirichlet head) are assigned with specified heads.

### Model calibration

**Fig. 13:**
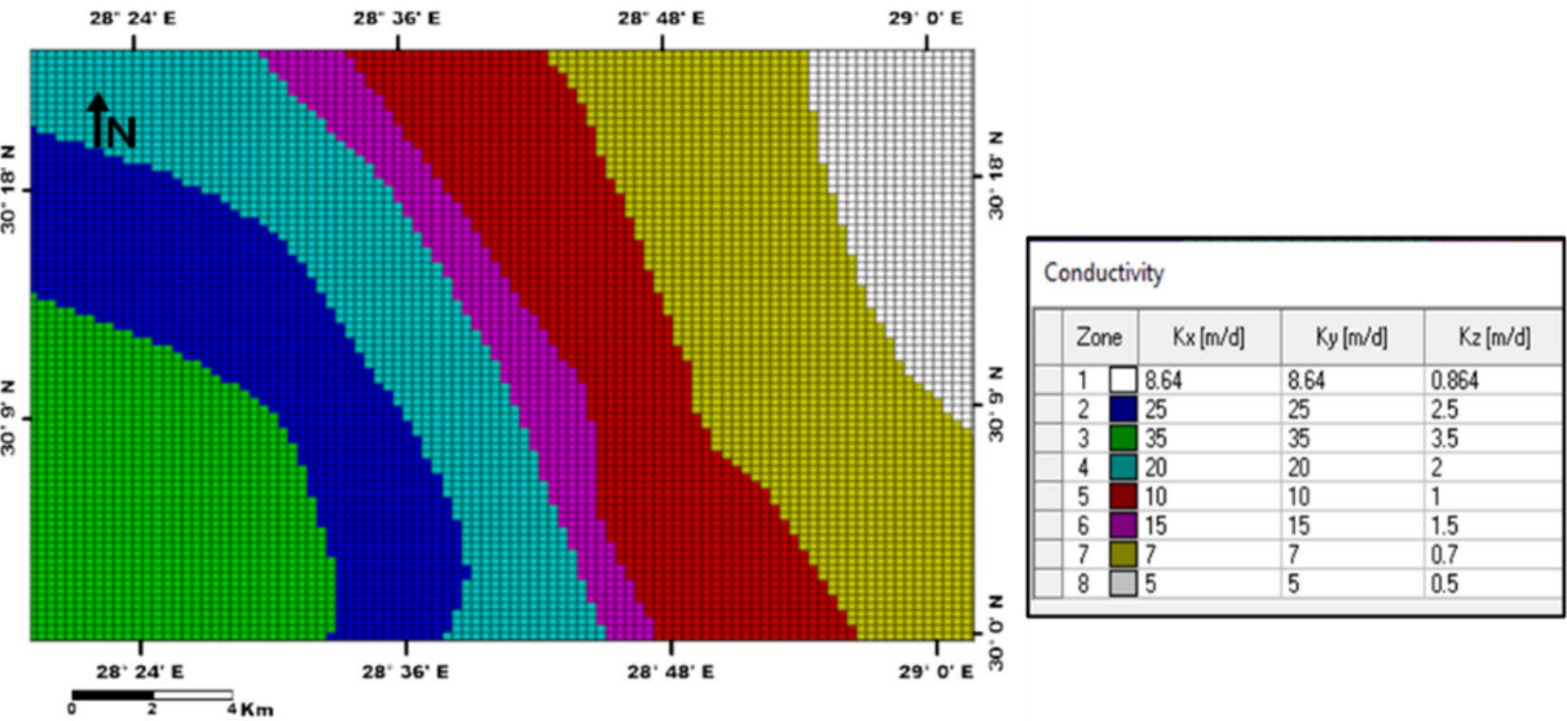
Calibrated hydraulic conductivity, Moghra aquifer, Western Desert, Egypt.

**Fig. 14:**
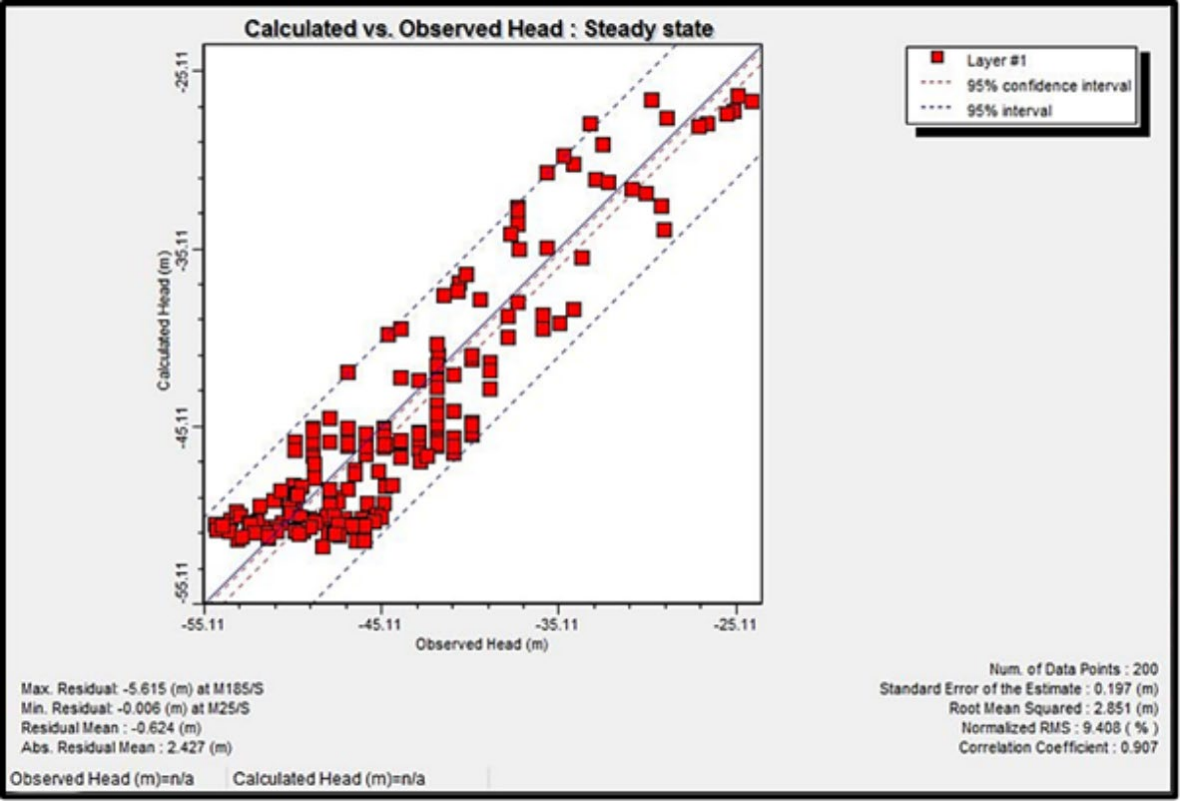
Calculated versus observed heads in the steady-state calibration.

**Fig. 15:**
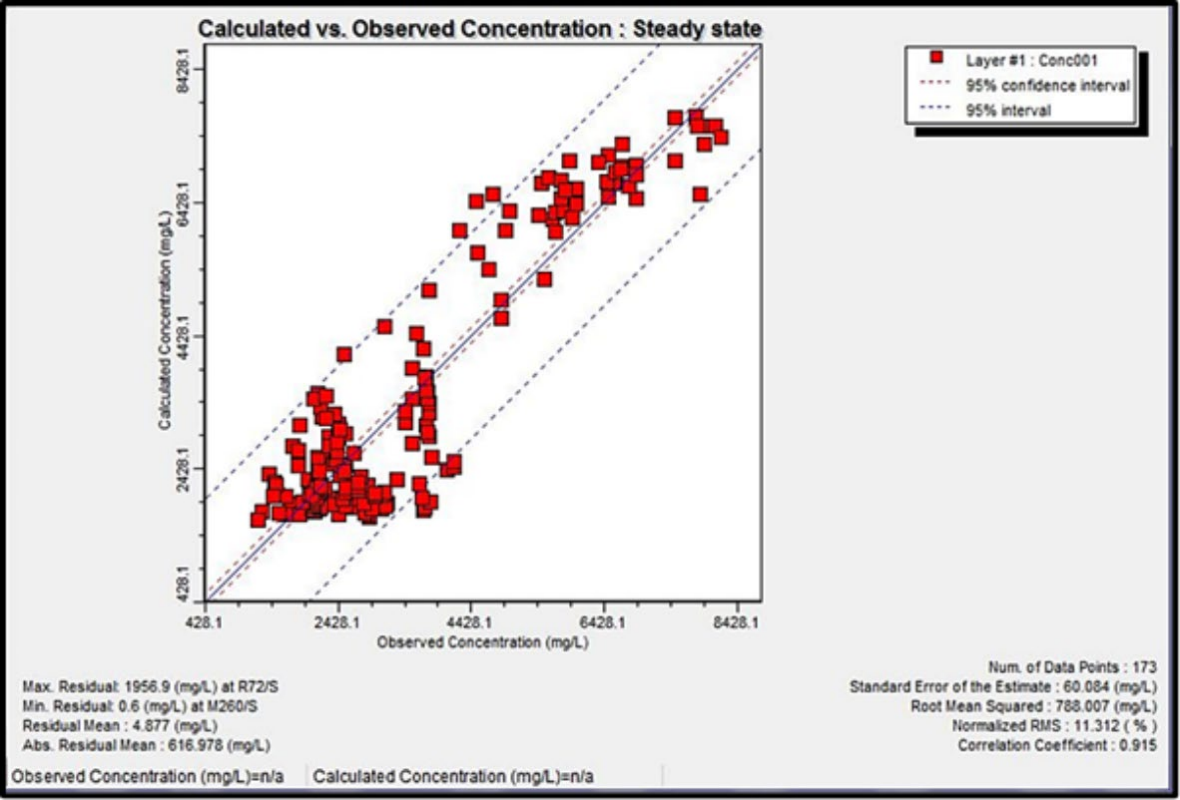
Calculated versus observed concentrations in the steady-state calibration.

The calibration process involves selecting aquifer parameter values that enable the model to simulate and produce heads and flows that acceptably match field-measured heads and flows for specified historic boundary conditions. At first, the model has undergone steady-state simulation with the initial heads for the MODFLOW module. Hydraulic conductivity values and boundary conditions were adjusted to minimize the difference between simulated and observed heads, or the so-called “calibration criterion” (Figs. 13 and 14). The solute transport calibration has been done using the initial solute concentration and the modification of effective porosity and longitudinal dispersivity (Fig. 15). After the steady-state simulation, the model is subjected to transient simulation for a short period to modify the Storativity values. Post-calibration sensitivity analysis involves more simulations. Before each simulation, a specific calibrated aquifer parameter or boundary condition is changed by a specific proportion. The sensitivity analysis revealed a high sensitivity of the model toward hydraulic conductivity, pumping rate, and specific storage (Ss). Based on the hypothesis of a vertical increase in solute concentration with depth, the aquifer layer is divided into three concentrations sub-layers. The uppermost layer is of current measured salinity values of 1,500 to 7,800 mg/l, while the other sublayers (2) and (3) have salinity concentrations of 15,000 and 20,000 mg/l, respectively.

#### Proposed scenarios

The developed MODFLOW model simulates the proposed management scenarios concerning the impacts of reclamation and climatic change on the Moghra aquifer. The current climatic data estimated from Rain Gauges and Satellite Observations are inputted to the model with Rainfall (PPT) of 73.9 mm/year and evapotranspiration (PET) of 96.7 mm/year. Four scenarios have been simulated by implementing 1500 pumping wells distributed with an equal space of 500 m and applying different practical strategies. Utilizing the recommendation of the Ministry of Irrigation and Water Resources^28^ that the largest drawdown should not exceed 1.0 m/ year, the first scenario has been simulated with a pumping rate of 1,200 m^3^/day. Two related scenarios have been simulated with higher and lower pumping rates than the first scenario: 1,600 and 800 m^3^/day. To adapt to the effects of climate change and groundwater depletion, the forecast of the climate records for 2020–2050 and the optimal pumping rate achieved are used to simulate an adaptation-integrated scenario.

## Results

### Climatic change impact on unconfined groundwater aquifer

**Fig. 16:**
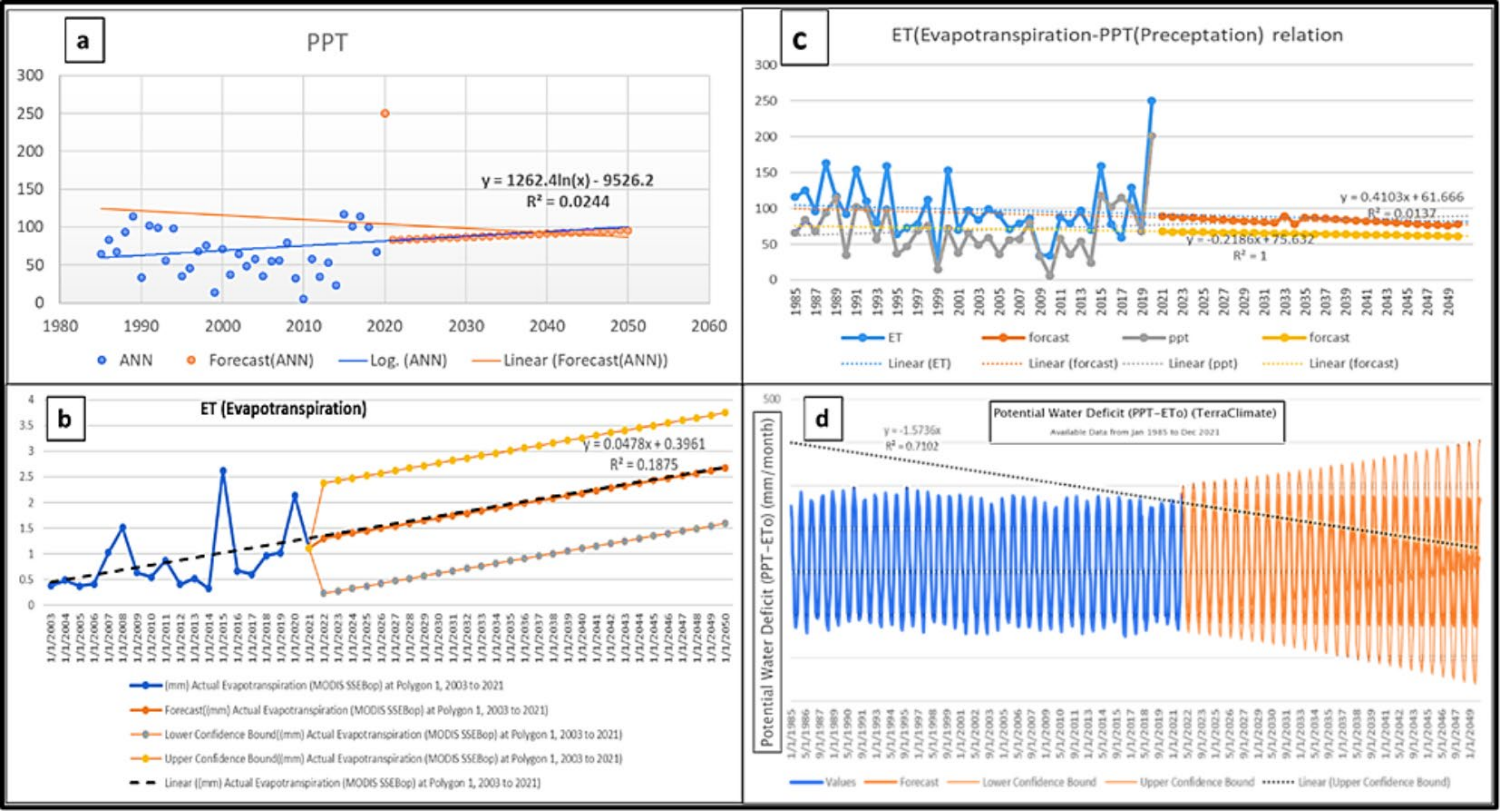
Statistical charts represent the long-term mean annual historical (1985 to 2021) and the forecast statistical value from (2022–2050). a): PPT rainfall precipitation, b): ET evapotranspiration, c): comparative relation between PPT and ET, and d): water deficit (PPT-ET).

Rainfall (PPT), Evaporation (ET), runoff, soil humidity, and temperature are the main factors that reflect water deficit processes influencing GWS variations due to water scarcity. Long-term ET rises from (1985–2020) with an average value of 96.88 mm/yr., when shallow groundwater supplies surface soil via capillary rise to satisfy increased Evaporation (ET) demand. But the ET forecast of (2020–2050) average value is about 159.72 mm/yr. The most significant climatic parameter impacting recharge was rainfall intensity; potential evapotranspiration and temperature also substantially impacted recharging. It is worth noting that changes in global climatic processes are predicted to cause a rise in average global temperature of 1.4 to 5.8 °C by the year 2100^35^. The Max annual temperature of the study area ranges from 25 °C in the north and increases toward the south reaching up to 30 °C, especially in the southwest which led to an increase the ET. The mean annual areal potential evapotranspiration for 37 long times from 1985 to 2022 varies from 56 mm in the north to 98 mm in the south. ET (evapotranspiration, the amount of both evaporation and transpiration) rises as atmospheric water demand rises. Rising temperatures cause slightly higher ET, which increases upward capillary flows from water to overlying soil moisture to fulfill the increasing atmospheric moisture demand. Long-term historical climatic data from meteorological stations show that the average annual rainfall ranges from 5.8 to 164 mm from 1985 to 2022 with an average value of about 73.99 mm/yr., but the forecast of PPT for 2020–2050 is with an average value of about 98.56 mm/yr. Rainfall from satellite data has a significant southwest-to-northeast gradient (Fig. 16a), with the maximum rainfall towards the northeast. Figure (16b) depicts the historical situation of evaporation and precipitation from 1985 to 2022 and the future forecast of the connection, which would result in increased water loss owing to increased evaporation. The soil moisture index (SMI) is defined as the ratio of the existing soil moisture to the permanent wilting limit to the soil surface and residual soil moisture. The index values vary from 0 to 1 with 0 representing severe dry environments and 1 indicating extreme moisture^33^. The soil moisture for the study area increased toward the northeast direction to reach (0.14). A gentle breeze and normal wind speed average from 10 to 30mph (The Beaufort Wind Scale, Royal Meteorological Society website), the maximum wind speed in the study area ranges from 2.8 to 3.32 m/sec in the gentle breeze category, increasing toward the north and southwest. The stream network density drains surface water (run-off) away to the north and northwest. The geological and climatic factors Influence the groundwater recharge and runoff harvesting patterns, perennial surface water flow areas frequently feature continuous infiltration and resultant groundwater flow. Run-off collection improves groundwater recharge facilities, particularly in dry environments. Groundwater recharge is estimated using the water balance approach as grid-cell precipitation minus real evapotranspiration^35^, runoff and recharge increased toward the north with the same direction of earth slope and wind speed direction (Fig. 16c). The direct relationship between climate change and the hydrological cycle, temperature changes, precipitation intensity, changes in precipitation quantity and seasonal distribution, increased evapotranspiration, and decreased soil moisture are all indicators of a sharp decline in the water table and a dramatic decrease in recharge. The drop in GWS is mostly due to the cumulative effects of over-pumping and climatic changes; nevertheless, the contribution of pumping might easily surpass natural resupply. Applying the projected future recharge (rainfall, runoff, max temperature, and Evaporation) time series as an input to a groundwater model and analysis of the water deficit. The highest and lowest GWS peaks were shown to shift in time with increasing aquifer depth relative to the maximum and minimum climatic parameters (PPT, Temp and ET) peaks (Fig. 16d). In future warming environments, time series analysis and forecast are critical for analyzing the data. The capacity to look forward and backward in time and efficiently and quickly make temporal comparisons such as year-over-year growth and moving averages. The average global temperature is expected to be from 1.4 to 5.8 °C by the year 2100 because of changes in global climatic processes^36^. The forecast of PPT for 2020–2050 is an average value of about 98.56 mm/yr. The mean annual areal potential evapotranspiration increased from north to south. When the mean annual rainfall deficit is calculated by subtracting actual potential evapotranspiration (APET) from rainfall, almost all study area have a negative water deficit over time.

### Simulation of the proposed scenarios

**Fig. 17:**
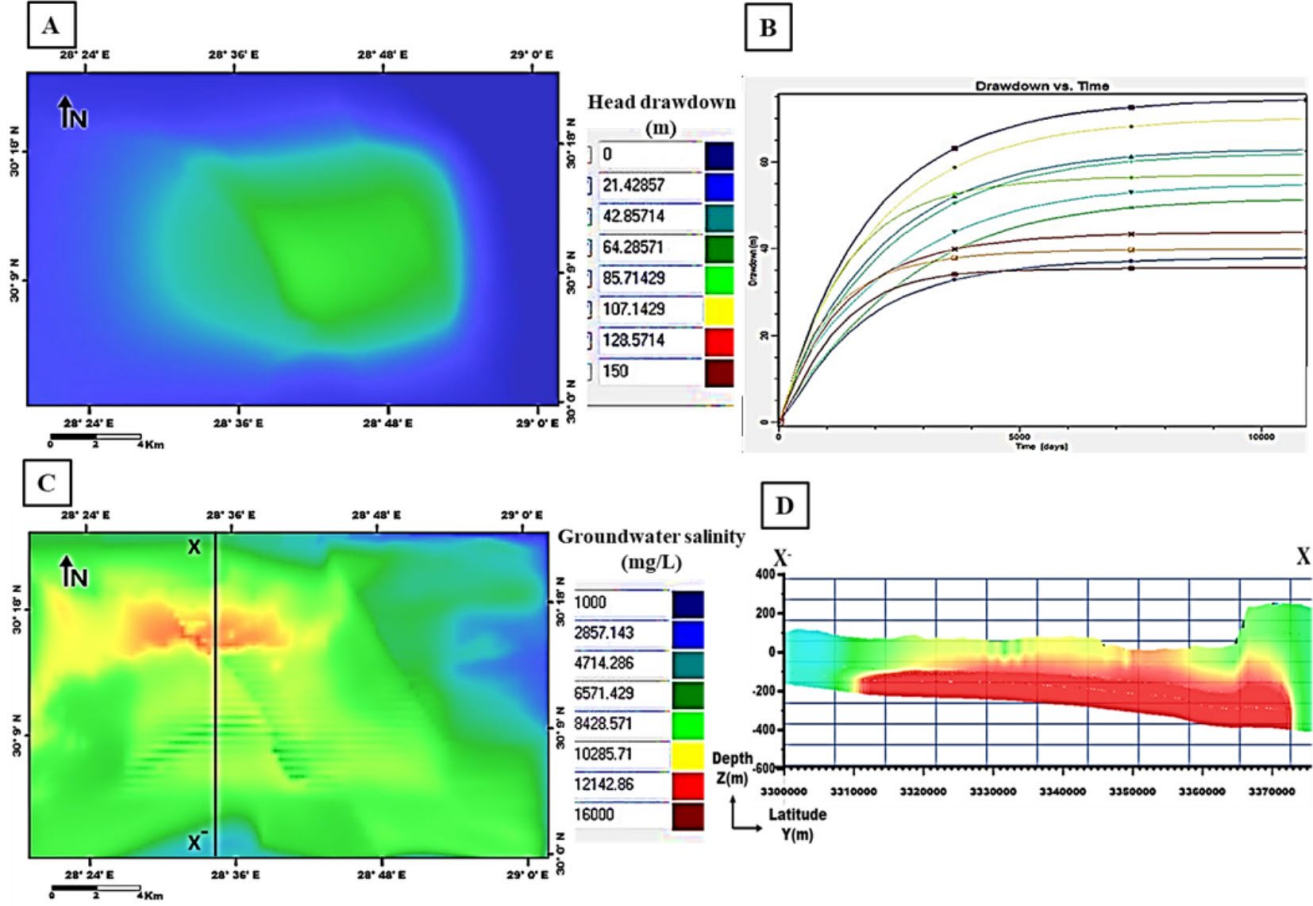
Output of the first scenario simulation: **A** Head drawdown, **B** Drawdown vs. time, **C** Groundwater salinity, and **D** Salinity distribution sub-layers along the cross-section X–X′ versus depth.

**Fig. 18:**
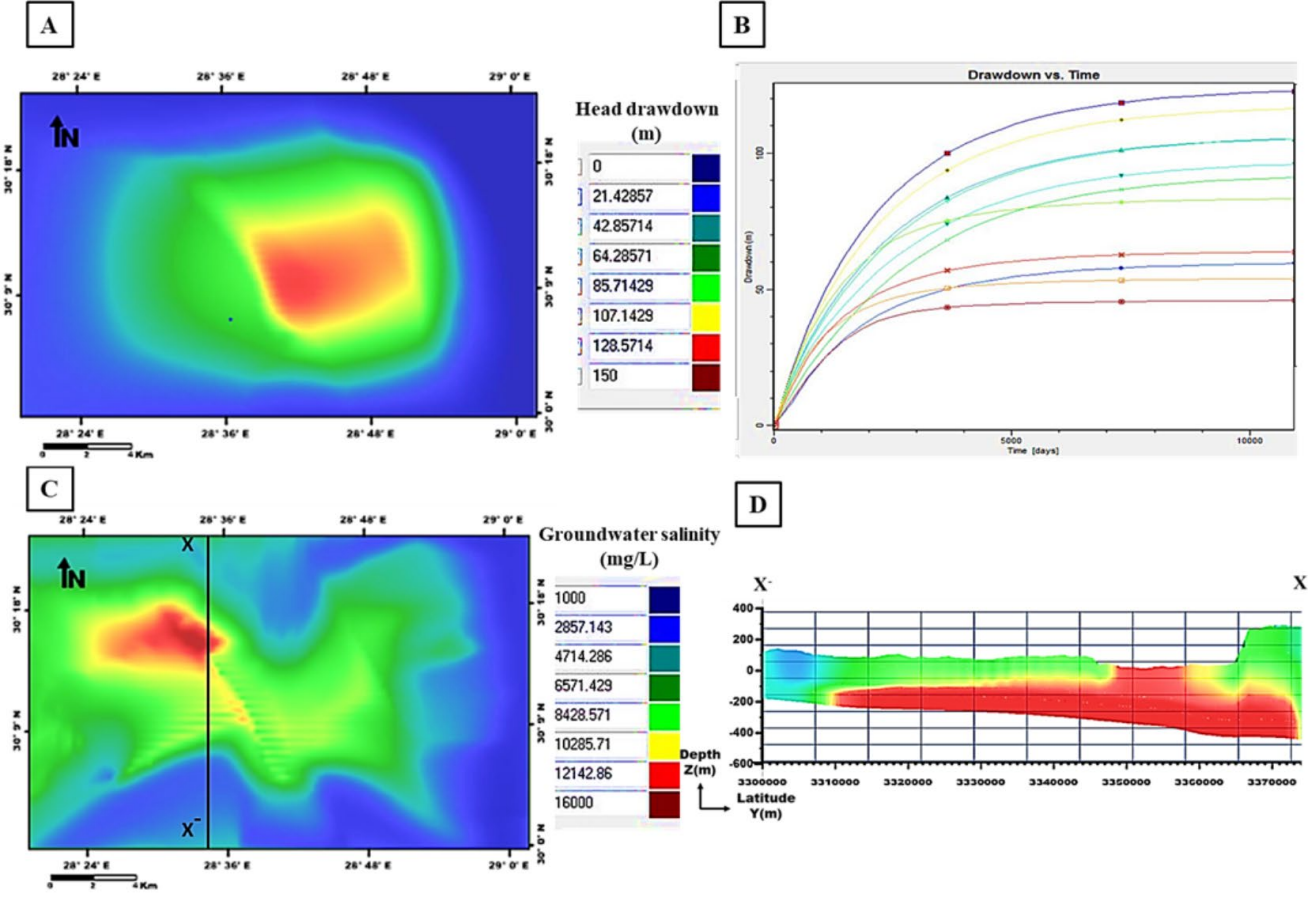
Output of the second scenario simulation: **A** Head drawdown, **B** Drawdown vs. time, **C** Groundwater salinity, and **D** Salinity distribution sub-layers along the cross-section X–X′ versus depth.

**Fig. 19:**
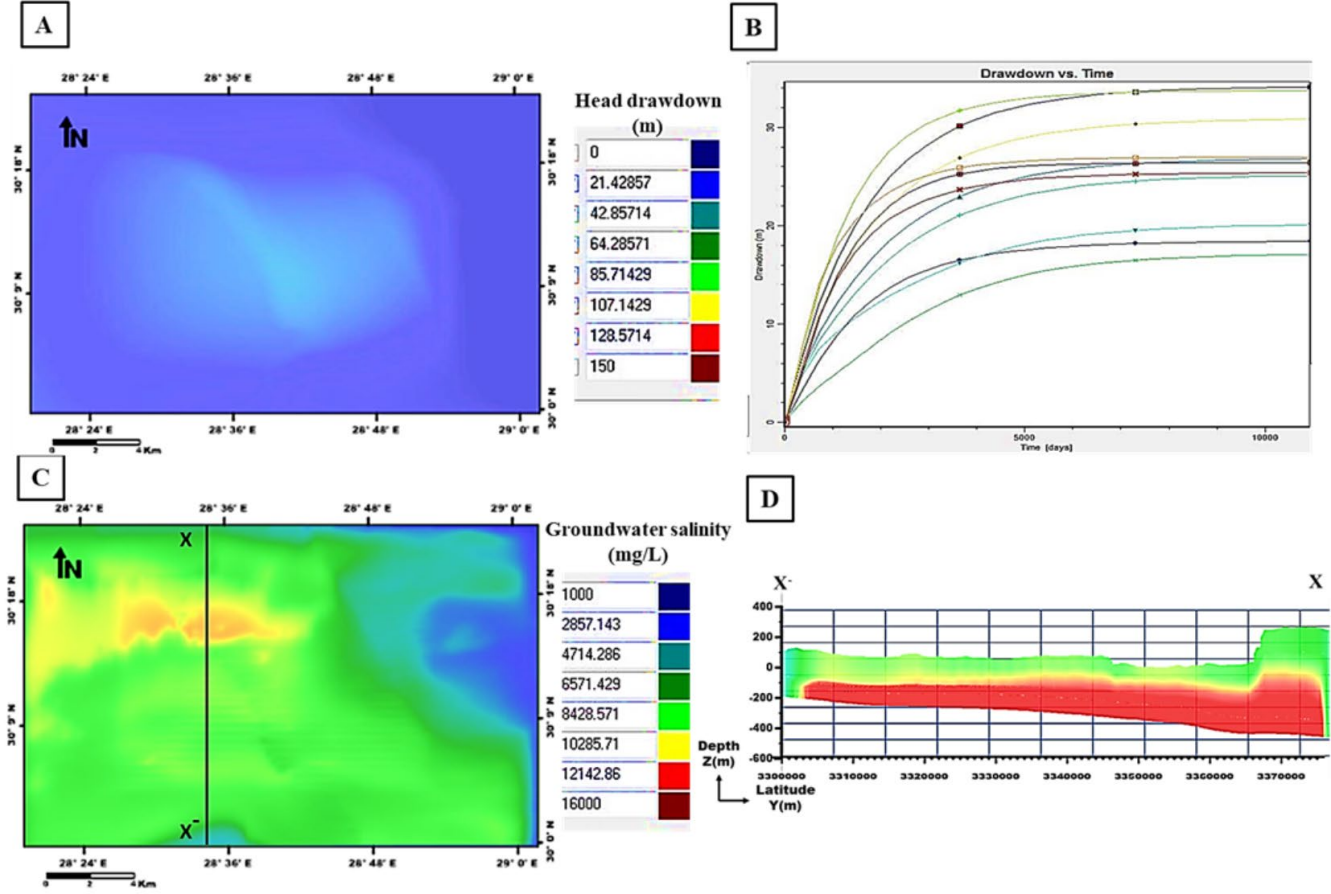
Output of the third scenario simulation: **A** Head drawdown, **B** Drawdown vs. time, **C** Groundwater salinity, and **D** Salinity distribution sub-layers along the cross-section X–X′ versus depth.

**Fig. 20:**
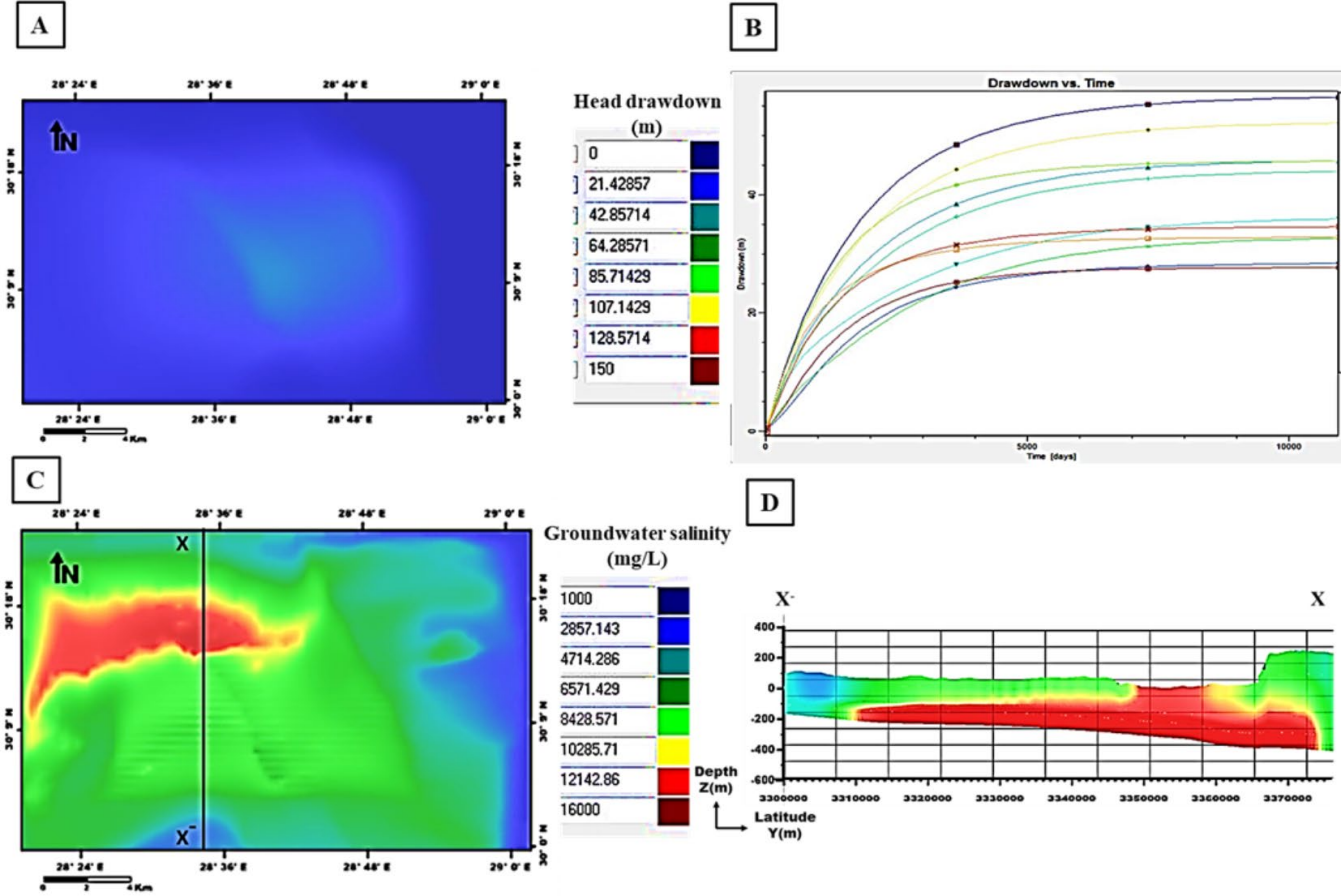
Output of the adaptation-integrated scenario simulation: **A** Head drawdown, **B** Drawdown vs. time, **C** Groundwater salinity, and **D** Salinity distribution sub-layers along the cross-section X–X′ versus depth.

The output of the first scenario simulation shows a decline in groundwater heads and an increase in salinity; recording a maximum drawdown of 85 m and groundwater salinity of 12,142 mg/l (Fig. 17). When increasing the pumping rate to 1600 m^3^/day in the second scenario, a noticeable increase in the drawdown resulted in 128 m with high salinity values of 14,000 mg/l (Fig. 18). By decreasing the pumping rate to 800 m^3^/day in the third scenario, the output drawdown reached 35 m, and salinity reached 10,285 mg/l (Fig. 19). The decision support for defining the optimal scenario is to meet the needs of the development area quantitatively or qualitatively without opportunities for groundwater degradation. Although the output results of the third scenario show the lowest levels of drawdown and groundwater salinity, they don’t keep up with the aquifer efficiency or the reclamation requirements. Therefore, the first scenario is considered optimal to achieve the best exploitation of the Moghra aquifer. the forecast downscaling (2020–2050) of main climatic parameters (PPT, ET, and Temp) used as input parameters with the value of PPT is 98.5 mm/year and of ET is 159.7 mm/year. The simulated adaptation-integrated scenario results revealed a drawdown of 42 m and salinity values of 16,000 mg/l, (Fig. 20). The expected recharge source to the Moghra aquifer from rainfall precipitation played an influential role in decreasing the drawdown level in this suggested scenario.

## Conclusions

The Egyptian government intended to expand the development of agriculture in the Moghra area, in the northwestern desert of Egypt. Hundreds of pumping wells were constructed in the Moghra aquifer to fulfil reclamation demands. Meeting the needs of the development area without opportunities for quantitative or qualitative groundwater degradation is a great challenge; specifically for the unconfined or shallow aquifers which are more sensitive to climate change and seawater intrusion. The present work introduces an unconventional approach by applying the MODFLOW and Solute Transport (MT3DMS) modules integrated with the predicted impact of climate change estimated using satellite data, time series geographical analysis, and statistical modelling. According to the forecast scenario, actual evapotranspiration (mm/year) ranges from 40 to 200 mm, with an average value of around 96.88 mm/yr. The simulation forecasts an increase in average evapotranspiration of 159.72 mm/yr for the province. (2.3%) and increase in PPT for 2020–2050 reaching an average value of about 98.56 mm/yr. The simulated optimal adaptation-integrated scenario shows a lower decline in groundwater heads. The increase in the forecast values of PPT plays a significant role in minimizing drawdown levels. The findings in this work reveal the high potentiality of the Moghra aquifer in long-term sustainable water development exclusively with the optimal exploitation of the groundwater.

## Electronic supplementary material

Below is the link to the electronic supplementary material.

Supplementary Material 1

## Data Availability

This article has no associated data, and all the data used in this study are present in the article.
