# Supplementary material for: Visual MODFLOW, solute transport modeling, and remote sensing techniques for adapting aquifer potentiality under reclamation and climate change impacts in coastal aquifer
Source: Sci Rep. 2024 Oct 1;14:22827. doi: 10.1038/s41598-024-72933-8 (PMC11445270; doi:10.1038/s41598-024-72933-8)
Supplement: Supplementary file 1 — Supplementary Material 1 [file 41598_2024_72933_MOESM1_ESM.docx]

| WELL ID | water level | Depth to water | TDS | Transmissivity | Draw down m3/hr |
| --- | --- | --- | --- | --- | --- |
| A102 | -37.33 | 33.5457 | 8650 | 1460 | 3.2568 |
| A103 | -23.4 | 14.07252 | 8650 | 1860 | 2.538 |
| A104 | -43.3 | 24.82344 | 8190 | 1290 | 4.2216 |
| A105 | -37.42 | 29.45245 | 8100 | 1750 | 2.9712 |
| A106 | -33.2 | 24.75292 | 7940 | 1720 | 2.7612 |
| A107 | -25.25 | 16.79558 | 7930 | 900 | 4.6236 |
| A108 | -19.6 | 5.367239 | 7876 | 1670 | 2.3148 |
| A112 | -41.5 | 44.86408 | 7830 | 427 | 11.8416 |
| A113 | -42.05 | 38.04576 | 7800 | 900 | 5.298 |
| A114 | -29.35 | 27.39355 | 7630 | 1860 | 2.538 |
| A115 | -26.2 | 33.29059 | 7490 | 530 | 10.2924 |
| A116 | -37.4 | 27.59749 | 7000 | 775 | 6.4524 |
| A117 | -39.62 | 18.57993 | 6900 | 1960 | 2.5572 |
| A118 | -28.5 | 9.070787 | 6900 | 1900 | 4.4196 |
| A119 | -29.2 | 22.63034 | 6900 | 916 | 5.352 |
| A120 | -41.6 | 22.12877 | 6800 | 8380 | 5.8248 |
| A121 | -32.42 | 28.4666 | 6700 | 2680 | 1.3908 |
| A122 | -32.7 | 36.05253 | 6680 | 596 | 8.4948 |
| A123 | -37.38 | 38.1189 | 6670 | 1260 | 4.0152 |
| A124 | -40.06 | 28.02007 | 6590 | 1110 | 4.122 |
| A125 | -33.02 | 13.56315 | 6580 | 961 | 4.8408 |
| A126 | -23.75 | 5.954276 | 6500 | 9120 | 4.9152 |
| A127 | -23.15 | 22.56102 | 6500 | 9390 | 5.07 |
| A128 | -37.8 | 12.6836 | 6470 | 525 | 9.5832 |
| A129 | -26.55 | 2.525488 | 6350 | 1520 | 2.898432 |
| A130 | -22.13 | 15.89953 | 6040 | 1260 | 4.4196 |
| A131 | -29.82 | 14.06377 | 6028 | 1440 | 3.438 |
| A132 | -29.55 | 48.3109 | 6026 | 327 | 12.7104 |
| A133 | -38.3 | 50.55194 | 6015 | 1820 | 2.456064 |
| A136 | -34.8 | 34.1037 | 6000 | 780 | 5.5644 |
| A138 | -33.33 | 36.13851 | 6000 | 835 | 5.3088 |
| A139 | -37.9 | 27.33273 | 6000 | 1270 | 4.3212 |
| A140 | -35.75 | 17.79068 | 5950 | 729 | 6.84288 |
| A141 | -30.96 | 24.57449 | 5943 | 1770 | 3.1884 |
| A142 | -37.4 | 6.351087 | 5905 | 1630 | 2.8908 |
| A143 | -21.69 | 2.47033 | 5900 | 731 | 7.6908 |
| A144 | -23.5 | 16.15567 | 5900 | 730 | 2.691072 |
| A145 | -25.86 | 13.53139 | 5880 | 1020 | 5.2464 |
| A146 | -28.2 | 7.037575 | 5840 | 2020 | 2.024064 |
| A147 | -23.12 | 59.54382 | 5820 | 909 | 4.5636 |
| A148 | -29.9 | 63.58169 | 5800 | 1890 | 1.865088 |
| A151 | -33.9 | 52.43421 | 5800 | 860 | 4.9008 |
| A152 | -39.8 | 45.35575 | 5780 | 770 | 5.1768 |
| A153 | -42.17 | 36.42051 | 5780 | 892 | 4.4292 |
| A154 | -32.65 | 29.46542 | 5780 | 855 | 4.2288 |
| A155 | -34.31 | 29.39109 | 5730 | 989 | 5.3496 |
| A156 | -32.32 | 12.34435 | 5700 | 1350 | 3.03552 |
| A157 | -25.37 | 9.988121 | 5700 | 731 | 7.6908 |
| A158 | -22.4 | 8.946584 | 5690 | 384 | 9.8316 |
| A159 | -24.4 | 17.83108 | 5664 | 1140 | 4.402944 |
| A160 | -29.5 | 19.84482 | 5650 | 328 | 6.77376 |
| A161 | -31.25 | 13.19849 | 5620 | 317 | 7.56288 |
| A162 | -25.84 | 81.87452 | 5620 | 768 | 7.0272 |
| A163 | -25 | 68.51626 | 5600 | 883 | 4.3536 |
| A164 | -24.2 | 79.1934 | 5550 | 568 | 5.8632 |
| A165 | -38.1 | 62.05928 | 5520 | 626 | 6.0108 |
| A166 | -37.45 | 49.89742 | 5490 | 473 | 8.3208 |
| A167 | -29 | 45.27092 | 5450 | 568 | 9.34272 |
| A168 | -26.75 | 71.58587 | 5450 | 724 | 5.562 |
| A169 | -49.6 | 40.55837 | 5400 | 651 | 6.2976 |
| A170 | -34.81 | 37.74053 | 5400 | 1190 | 3.3852 |
| A171 | -36.21 | 26.16385 | 5400 | 1650 | 2.6904 |
| A172 | -30.22 | 27.5958 | 5400 | 1010 | 4.3488 |
| A173 | -37.4 | 19.16748 | 5397 | 568 | 6.794496 |
| A174 | -29.17 | 34.1025 | 5350 | 594 | 7.3068 |
| A175 | -35.76 | 43.66611 | 5250 | 713 | 4 |
| A176 | -43.1 | 47.5747 | 5240 | 1090 | 3.84 |
| A67 | -40.75 | 34.18722 | 5200 | 1270 | 3.125376 |
| A68 | -38 | 51.28844 | 5090 | 461 | 8.395776 |
| A71 | -41.9 | 26.27551 | 5090 | 1270 | 2.9472 |
| A72 | -56.3 | 54.46104 | 5080 | 1610 | 2.082 |
| A73 | -72.8 | 75.31836 | 5060 | 1030 | 3.5292 |
| A75 | -71 | 18.89915 | 4998 | 1360 | 2.927232 |
| A76 | -48.8 | 30.99505 | 4990 | 942 | 3.9828 |
| A77 | -35.1 | 49.1982 | 4980 | 1390 | 3.071232 |
| A78 | -54.2 | 12.8707 | 4950 | 2180 | 1.9236 |
| A79 | -26 | 19.78508 | 4950 | 606 | 6.5208 |
| A80 | -27.2 | 20.70385 | 4900 | 1050 | 3.7884 |
| A81 | -39.53 | 34.26252 | 4890 | 1030 | 3.7068 |
| A82 | -38 | 34.98264 | 4890 | 2750 | 1.4808 |
| A83 | -44.77 | 30.9024 | 4870 | 1850 | 1.88352 |
| A84 | -35.33 | 27.72228 | 4850 | 906 | 4.6392 |
| A87 | -34 | 31.59762 | 4840 | 833 | 6.732 |
| A88 | -33.8 | 36.13649 | 4810 | 642 | 11.9244 |
| A89 | -40.3 | 31.82707 | 4810 | 1290 | 2.7612 |
| A90 | -40.8 | 29.95935 | 4800 | 1770 | 2.0472 |
| A91 | -34.26 | 16.81521 | 4800 | 1850 | 2.3028 |
| A92 | -30.3 | 21.19578 | 4800 | 833 | 6.0108 |
| A93 | -25.63 | 17.77991 | 4800 | 2300 | 1.8336 |
| A94 | -26 | 20.04437 | 4800 | 1050 | 3.7884 |
| M100 | -36.17 | 54.10297 | 4800 | 4150 | 3.70752 |
| M115 | -36 | 53.83833 | 4800 | 2840 | 2.65248 |
| M130 | -40.5 | 68.95648 | 4800 | 6270 | 1.68624 |
| M132 | -44.75 | 67.77994 | 4780 | 1940 | 11.028 |
| M134 | -31 | 63.40931 | 4750 | 4650 | 5.712 |
| M135 | -49 | 31.14145 | 4750 | 4650 | 4.308 |
| M136 | -32 | 32.74863 | 4750 | 4450 | 4.176 |
| M149 | -36 | 54.34394 | 4750 | 4850 | 3.768 |
| M152 | -40 | 78.50214 | 4750 | 1310 | 11.196 |
| M153 | -42 | 72.43018 | 4750 | 3680 | 5.712 |
| M154 | -42 | 25.10602 | 4730 | 4150 | 4.4592 |
| M155 | -21 | 26.05886 | 4720 | 4550 | 4.308 |
| M156 | -33 | 27.97278 | 4720 | 4250 | 3.92448 |
| M157 | -47 | 36.91385 | 4710 | 5240 | 5.5968 |
| M167 | -44 | 83.8088 | 4700 | 1920 | 9.4032 |
| M168 | -42 | 74.17449 | 4700 | 2180 | 9.1704 |
| M169 | -41 | 57.67052 | 4690 | 2260 | 10.38 |
| M170 | -32 | 72.06668 | 4680 | 6990 | 3.66048 |
| M171 | -55 | 37.79451 | 4670 | 3150 | 4.272 |
| M172 | -37 | 6.110229 | 4670 | 4040 | 3.564 |
| M173 | -23 | 35.97342 | 4640 | 4480 | 4.716 |
| M174 | -41 | 62.57923 | 4620 | 3730 | 4.356 |
| M184 | -49 | 75.47488 | 4600 | 1880 | 10.548 |
| M185 | -45 | 59.50118 | 4600 | 1710 | 8.6592 |
| M186 | -40 | 54.25781 | 4600 | 3010 | 5.42352 |
| M187 | -42 | 69.47757 | 4570 | 5120 | 3.64848 |
| M188 | -45 | 77.38468 | 4567 | 1620 | 10.8888 |
| M189 | -43 | 64.54079 | 4567 | 1650 | 11.13648 |
| M190 | -32 | 59.99863 | 4560 | 2000 | 11.3736 |
| M191 | -36 | 47.67359 | 4560 | 3010 | 5.42352 |
| M192 | -39 | 56.96947 | 4560 | 3140 | 6.5232 |
| M203 | -49 | 61.87416 | 4550 | 2310 | 5.796 |
| M204 | -37 | 69.13128 | 4550 | 1040 | 5.90352 |
| M205 | -43 | 59.41663 | 4540 | 2130 | 5.19168 |
| M206 | -35 | 64.27564 | 4540 | 1390 | 11.2152 |
| M207 | -43 | 67.88351 | 4540 | 3220 | 3.67968 |
| M208 | -40 | 72.9153 | 4530 | 1450 | 10.9992 |
| M209 | -43 | 64.44765 | 4520 | 6850 | 4.04352 |
| M210 | -45 | 56.04641 | 4520 | 3590 | 4.12752 |
| M211 | -39 | 57.70417 | 4520 | 3910 | 5.232 |
| M212 | -41 | 55.52066 | 4520 | 5120 | 5.41152 |
| M218 | -46 | 69.32015 | 4500 | 4480 | 4.46352 |
| M223 | -49 | 68.0808 | 4500 | 3530 | 5.6952 |
| M224 | -49 | 67.757 | 4500 | 4000 | 5.628 |
| M225 | -45 | 61.2487 | 4500 | 4520 | 7.32 |
| M226 | -42 | 64.47133 | 4500 | 1950 | 8.8632 |
| M227 | -44 | 64.02006 | 4500 | 3690 | 6.0768 |
| M228 | -49 | 54.30017 | 4500 | 4180 | 7.064407 |
| M229 | -42 | 42.215 | 4500 | 2940 | 8.3328 |
| M230 | -20 | 33.66214 | 4498 | 2060 | 7.04448 |
| M231 | -23 | 51.53888 | 4490 | 3050 | 5.41152 |
| M241 | -45 | 71.66212 | 4480 | 5860 | 4.46352 |
| M242 | -55 | 68.41344 | 4480 | 3530 | 5.748 |
| M243 | -53 | 61.1744 | 4460 | 3570 | 9.0648 |
| M244 | -50 | 60.26796 | 4460 | 3720 | 5.54448 |
| M245 | -54 | 50.32428 | 4450 | 3630 | 4.60848 |
| M246 | -52 | 45.58377 | 4450 | 4370 | 4.46352 |
| M25 | -46 | 25.08772 | 4450 | 4990 | 3.62448 |
| M254 | -46 | 57.18352 | 4432 | 3080 | 5.42352 |
| M256 | -44 | 46.26275 | 4430 | 5260 | 3.816 |
| M257 | -35 | 51.11306 | 4425 | 3690 | 5.5968 |
| M258 | -43 | 42.01618 | 4420 | 4200 | 5.3712 |
| M259 | -47 | 43.21754 | 4420 | 4240 | 3.712752 |
| M260 | -48 | 35.36922 | 4420 | 4440 | 4.03248 |
| M268 | -39 | 54.85847 | 4410 | 3580 | 5.87232 |
| M269 | -47 | 54.54854 | 4400 | 5120 | 5.34048 |
| M272 | -35 | 39.51775 | 4400 | 7010 | 2.55552 |
| M273 | -45 | 39.63508 | 4400 | 1010 | 1.94208 |
| M274 | -49 | 52 | 4400 | 7340 | 3.012 |
| M275 | -57 | 44.98934 | 4400 | 4310 | 8.1048 |
| M276 | -55 | 43.97982 | 4390 | 4970 | 4.76736 |
| M277 | -53 | 47.07174 | 4390 | 3900 | 5.232 |
| M282 | -46 | 40.88752 | 4380 | 3590 | 5.28336 |
| M283 | -36 | 54.41525 | 4365 | 4400 | 4.9128 |
| M284 | -43 | 52.42332 | 4365 | 3240 | 5.14752 |
| M285 | -39 | 37.42013 | 4360 | 4330 | 3.94752 |
| M288 | -44 | 40.93366 | 4350 | 4430 | 4.572 |
| M289 | -50 | 37.56187 | 4350 | 4580 | 4.38048 |
| M290 | -46 | 33.99785 | 4345 | 4330 | 3.94752 |
| M291 | -40 | 43.66216 | 4343 | 4410 | 3.94752 |
| M292 | -41 | 48.87172 | 4340 | 4040 | 4.056 |
| M294 | -50 | 33.38504 | 4340 | 4460 | 4.46352 |
| M295 | -46 | 42.51548 | 4326 | 4250 | 4.42752 |
| M296 | -57 | 30.9134 | 4326 | 4450 | 4.50384 |
| M298 | -43 | 25.02465 | 4321 | 5900 | 2.298 |
| M299 | -33 | 27.01613 | 4320 | 4270 | 3.81072 |
| M300 | -31 | 36.74332 | 4320 | 4230 | 3.66048 |
| M301 | -42 | 36.78684 | 4320 | 4200 | 3.51552 |
| M303 | -42 | 28.71288 | 4320 | 4450 | 3.64848 |
| M304 | -47 | 26.04873 | 4320 | 4140 | 4.6008 |
| M305 | -43 | 27.93552 | 4320 | 6090 | 2.31552 |
| M306 | -42 | 31.91286 | 4320 | 4290 | 3.312 |
| M307 | -39 | 40.2772 | 4320 | 3790 | 2.84832 |
| M308 | -49 | 37.98885 | 4320 | 4560 | 3.91056 |
| M314 | -34 | 23.6465 | 4320 | 3130 | 6.5568 |
| M317 | -40 | 22.21132 | 4320 | 4220 | 2.822448 |
| M318 | -46 | 14.47907 | 4320 | 6200 | 3.912 |
| M319 | -31 | 20.85964 | 4312 | 7910 | 1.795675 |
| M320 | -40 | 31.38494 | 4312 | 5900 | 2.41752 |
| M325 | -39 | 31.41407 | 4310 | 3310 | 6.8112 |
| M326 | -42 | 32.20478 | 4310 | 4690 | 4.404 |
| M327 | -41 | 34.78544 | 4310 | 4500 | 4.728 |
| M328 | -48 | 20.72482 | 4310 | 4040 | 3.50352 |
| M329 | -42 | 14.18672 | 4280 | 7450 | 2.07552 |
| M330 | -37 | 15.27321 | 4270 | 5620 | 2.62032 |
| M331 | -47 | 31.74548 | 4250 | 3890 | 3.1968 |
| M333 | -46 | 5.030923 | 4250 | 6350 | 3.228 |
| M334 | -16 | 34.27372 | 4250 | 4050 | 3.50352 |
| M335 | -44 | 11.91739 | 4250 | 5620 | 2.53152 |
| M336 | -47 | 32.1948 | 4248 | 5900 | 2.48952 |
| M337 | -44 | 17.60114 | 4230 | 4230 | 3.04032 |
| M338 | -32 | 19.80082 | 4230 | 4250 | 3.312 |
| M345 | -33 | 31.63213 | 4230 | 5120 | 3.6144 |
| M346 | -42 | 35.19993 | 4200 | 6220 | 3.68448 |
| M347 | -56 | 21.57003 | 4200 | 4780 | 2.832 |
| M348 | -47 | 15.33173 | 4200 | 5460 | 3.1008 |
| M349 | -45 | 6.340206 | 4200 | 5960 | 3.50352 |
| M350 | -23 | 14 | 4200 | 4940 | 2.03952 |
| M353 | -40 | 26.19031 | 4200 | 4200 | 3.43152 |
| M354 | -43 | 12.91999 | 4200 | 4380 | 4.86 |
| M355 | -47 | 36.01425 | 4190 | 4340 | 3.24864 |
| M364 | -51 | 15.29956 | 4180 | 2250 | 3.29568 |
| M365 | -31 | 7.204419 | 4180 | 6040 | 2.93568 |
| M366 | -34 | 31.87595 | 4180 | 6340 | 3.42048 |
| M367 | -54 | 12.05957 | 4170 | 4970 | 2.29968 |
| M368 | -37 | 13.95701 | 4170 | 6130 | 3.312 |
| M369 | -30 | 16.59415 | 4150 | 4910 | 1.92336 |
| M371 | -45 | 16.36261 | 4150 | 5730 | 2.70048 |
| M372 | -49 | 14.41981 | 4150 | 5600 | 3.01152 |
| M373 | -51 | 1.672764 | 4150 | 2100 | 4.085904 |
| M374 | -46 | 1.384767 | 4140 | 1710 | 10.60584 |
| M375 | -45 | 23.60849 | 4130 | 4420 | 3.36384 |
| M377 | -36 | 26.56551 | 4108 | 4840 | 2.832 |
| M384 | -42 | 23.10632 | 4105 | 5260 | 6.85152 |
| M386 | -50 | 18.63898 | 4100 | 6220 | 3.792 |
| M394 | -40 | 9.6399 | 4100 | 3580 | 3.57408 |
| M396 | -21 | 28.61899 | 4100 | 4290 | 3.03552 |
| M397 | -34 | 16.37951 | 4100 | 4910 | 2.88864 |
| M398 | -33 | 17.73589 | 4100 | 4290 | 2.30352 |
| M99 | -34 | 64.01466 | 4100 | 3430 | 2.7012 |
| R60 | -45 | 26.58095 | 3999 | 317 | 7.9704 |
| X10 | -46.6 | 40.12328 | 3890 | 3012 | 0.164972 |
| X100 | -46.6 | 68.03457 | 3882 | 832 | 6.3216 |
| X106 | -39.22 | 58.22516 | 3880 | 1214 | 5.0028 |
| X107 | -45.25 | 60.53603 | 3865 | 2421 | 0.08678 |
| X108 | -53.5 | 62.5719 | 3865 | 2242 | 1.138396 |
| X109 | -44.9 | 56.6044 | 3850 | 3511 | 0.117102 |
| X11 | -42.9 | 33.71035 | 3840 | 1454 | 0.191669 |
| X110 | -48.9 | 58.88506 | 3810 | 4100 | 4.8972 |
| X111 | -52.35 | 43.74012 | 3800 | 1838 | 0.177108 |
| X113 | -54.9 | 38.36466 | 3792 | 2362 | 0.124562 |
| X114 | -42.5 | 62.90116 | 3792 | 2294 | 0.183507 |
| X118 | -24 | 66.85555 | 3789 | 2414 | 0.135193 |
| X119 | -43 | 56.15461 | 3786 | 1276 | 3.766896 |
| X12 | -21.5 | 38.17431 | 3786 | 1075 | 0.147096 |
| X120 | 0 | 61.49655 | 3780 | 1253 | 5.1264 |
| X121 | -18 | 71.21817 | 3780 | 1233 | 0.148836 |
| X122 | -17 | 58.2673 | 3774 | 1264 | 0.154782 |
| X123 | -42.3 | 62.9572 | 3770 | 1481 | 0.126264 |
| X124 | -44.5 | 65.21538 | 3768 | 1119 | 4.2948 |
| X125 | -47 | 65.14083 | 3762 | 978 | 0.09568 |
| X126 | -40 | 68.0963 | 3756 | 954 | 0.176986 |
| X129 | -51 | 49.43903 | 3750 | 1430 | 0.110995 |
| X13 | -45.9 | 43.50078 | 3750 | 3768 | 6.516265 |
| X130 | -52 | 57.7918 | 3744 | 898 | 5.43 |
| X131 | -50 | 53.19264 | 3732 | 2498 | 6.1704 |
| X132 | -48.9 | 61.91185 | 3726 | 2934 | 6.69 |
| x133 | -41.8 | 67.22589 | 3720 | 735 | 10.95 |
| X134 | -39.2 | 45.81193 | 3714 | 569 | 8.9568 |
| X135 | -50.1 | 66.7644 | 3700 | 735 | 0.171396 |
| X136 | -47.72 | 54.78997 | 3700 | 929 | 0.19775 |
| X137 | -47.6 | 66.67492 | 3690 | 2051 | 0.173856 |
| X138 | -48 | 37.82486 | 3645 | 1172 | 0.142974 |
| X139 | -38.7 | 69.23926 | 3600 | 968 | 0.192623 |
| X14 | -50.1 | 45.79397 | 3580 | 1595 | 0.180955 |
| X140 | -48 | 67.69417 | 3560 | 730 | 4.6824 |
| X141 | -49.9 | 62.19432 | 3540 | 1595 | 0.180955 |
| X142 | -51.2 | 76.96365 | 3534 | 1157 | 0.206448 |
| X143 | -45 | 33.5851 | 3534 | 1210 | 0.198181 |
| X144 | -50.3 | 37.3753 | 3534 | 1645 | 0.137966 |
| X145 | -40.7 | 73.26777 | 3490 | 1671 | 0.066876 |
| X146 | -49.6 | 50.52096 | 3470 | 2339 | 0.099651 |
| X147 | -50.8 | 36.89997 | 3470 | 1090 | 8.113584 |
| X148 | -49.85 | 58.23332 | 3470 | 758 | 0.444648 |
| X149 | -41.27 | 61.1341 | 3470 | 874 | 5.454 |
| X15 | -38.75 | 31.98013 | 3470 | 1169 | 4.8708 |
| X150 | -37.8 | 47.75692 | 3444 | 1452 | 6.4752 |
| X151 | -20.1 | 55.99282 | 3440 | 1245 | 0.152082 |
| X151" | -47.5 | 68.14001 | 3438 | 1789 | 0.130339 |
| X152 | -42.7 | 59.56812 | 3310 | 2125 | 0.10459 |
| X154 | -36.2 | 53.24199 | 3300 | 1983 | 0.189954 |
| X156 | -30.6 | 50.78238 | 3280 | 2294 | 0.149424 |
| X16 | -49.2 | 39.32623 | 3270 | 2420 | 0.162728 |
| X166 | -48.8 | 65.65258 | 3158 | 1410 | 4.8972 |
| X167 | -44.1 | 57.62634 | 3140 | 2861 | 0.13865 |
| X168 | -32.9 | 50.58456 | 3132 | 1650 | 0.186021 |
| X169 | -19.3 | 68.19208 | 3120 | 1202 | 0.178502 |
| X17 | -54.5 | 43.34754 | 3102 | 3135 | 0.149511 |
| X170 | -42.1 | 57.71905 | 3090 | 1253 | 5.1264 |
| X171 | -27.1 | 68.48707 | 3084 | 1602 | 0.1679 |
| X172 | -48.1 | 67.57967 | 2970 | 1722 | 0.19649 |
| X178 | -43.7 | 76.84169 | 2970 | 1508 | 0.140535 |
| X18 | -45.3 | 45.80553 | 2940 | 2339 | 0.099638 |
| X182 | -37.1 | 73.26891 | 2900 | 1255 | 4.8108 |
| X183 | -48.2 | 74.95894 | 2888 | 2991 | 0.174333 |
| X184 | -33 | 72.10593 | 2880 | 1837 | 0.870006 |
| X185 | -42.2 | 46.92306 | 2844 | 3707 | 0.988601 |
| X186 | -40.5 | 47.16311 | 2842 | 1293 | 8.4036 |
| X187 | -46.25 | 46.28694 | 2836 | 974 | 0.101066 |
| x188 | -31.75 | 32.50119 | 2820 | 1331 | 0.617184 |
| X189 | -42 | 31.2706 | 2766 | 1113 | 0.131256 |
| X19 | -50.71 | 46.78303 | 2760 | 1087 | 1.064914 |
| X190 | -42.25 | 65.01806 | 2736 | 898 | 8.388 |
| X191 | -41.75 | 63.6978 | 2736 | 1671 | 0.066876 |
| X192 | -36.4 | 44.27553 | 2736 | 1209 | 0.144359 |
| X193 | -48.1 | 55.13353 | 2724 | 2240 | 0.209832 |
| X194 | -38.8 | 78.26221 | 2670 | 1567 | 0.106179 |
| X195 | -38.8 | 84.54496 | 2652 | 3488 | 0.186381 |
| X196 | -45.37 | 88.04436 | 2640 | 1708 | 0.207636 |
| X197 | -53.3 | 57.79473 | 2560 | 1963 | 0.138503 |
| X198 | -31.95 | 62.36411 | 2550 | 2678 | 0.148988 |
| X199 | -47.2 | 41.63031 | 2544 | 1429 | 0.137439 |
| X2 | -39.15 | 34.70998 | 2538 | 1255 | 0.181181 |
| X20 | -43.9 | 40.25289 | 2532 | 1103 | 6.1536 |
| X200 | -18.9 | 56.10739 | 2532 | 953 | 5.298 |
| X201 | -48.8 | 35.32002 | 2532 | 898 | 8.388 |
| X202 | -47.8 | 43.4136 | 2520 | 1255 | 4.8108 |
| X203 | -46.3 | 35.45492 | 2520 | 953 | 1.98012 |
| X204 | -40.1 | 43.55688 | 2508 | 898 | 0.153454 |
| X21 | -51.8 | 52.57606 | 2496 | 1143 | 0.097719 |
| X213 | -17.5 | 34.42741 | 2460 | 1143 | 7.5684 |
| X22 | -23.6 | 47.73815 | 2440 | 3980 | 0.163853 |
| X221 | -58.6 | 51.52817 | 2434 | 1254 | 4.4556 |
| X222 | -38.92 | 51.18558 | 2430 | 1671 | 0.066876 |
| X223 | -28.75 | 46.58924 | 2424 | 1671 | 3.8196 |
| X224 | -47.8 | 28.7319 | 2421 | 944 | 4.3212 |
| X225 | -45.1 | 37.85692 | 2400 | 832 | 0.148331 |
| X227 | -34.55 | 43.63423 | 2370 | 1671 | 0.066876 |
| X23 | -35.75 | 32.07449 | 2349 | 1671 | 0.066876 |
| X231 | -41 | 55.32972 | 2342 | 622 | 8.6796 |
| X233 | -43.72 | 61.18953 | 2340 | 2780 | 0.160102 |
| X234 | -38.1 | 49.5959 | 2320 | 1424 | 0.152957 |
| X235 | -34.12 | 50.20438 | 2310 | 1318 | 0.177108 |
| X236 | -31.3 | 46.25008 | 2292 | 876 | 0.129629 |
| X237 | -44.44 | 44.3989 | 2292 | 944 | 0.15954 |
| X238 | -45.5 | 32.75327 | 2252 | 1254 | 0.107388 |
| X239 | -38.1 | 41.57173 | 2250 | 622 | 0.221808 |
| X24 | -37.9 | 40.67348 | 2240 | 1177 | 0.21727 |
| X240 | -46.6 | 38.47275 | 2220 | 1177 | 7.8672 |
| x241 | -45.75 | 34.7205 | 2178 | 1107 | 0.133883 |
| X242 | -39.1 | 41.85217 | 2172 | 1090 | 6.9516 |
| X243 | -47.39 | 38.56652 | 2166 | 1198 | 0.130389 |
| X244 | -42.9 | 47.1 | 2166 | 1178 | 0.121284 |
| X245 | -53.6 | 25.71962 | 2158 | 832 | 0.148331 |
| X246 | -46.52 | 46.12823 | 2154 | 3012 | 0.108948 |
| X247 | -46.1 | 45.59072 | 2154 | 944 | 0.15954 |
| X248 | -46.8 | 33.97892 | 2142 | 631 | 0.228747 |
| X249 | -44.5 | 44.43026 | 2142 | 735 | 0.136535 |
| X25 | -58.2 | 38.27809 | 2142 | 1143 | 0.097719 |
| X250 | -53.3 | 42.279 | 2138 | 944 | 4.7208 |
| X251 | -50.2 | 39.65257 | 2124 | 953 | 0.170412 |
| X252 | -56.9 | 37.89506 | 2124 | 898 | 1.12363 |
| X253 | -53.1 | 39.56298 | 2122 | 1198 | 0.130389 |
| X254 | -51.07 | 48.02802 | 2112 | 1827 | 0.206059 |
| X255 | -59.7 | 37.65263 | 2100 | 1214 | 0.097719 |
| X256 | -49.7 | 32.82851 | 2094 | 2492 | 0.032001 |
| X257 | -25.3 | 35.02283 | 2094 | 1647 | 0.808636 |
| X26 | -35.8 | 47.97761 | 2076 | 631 | 1.156228 |
| X29 | -52.1 | 39.26702 | 2072 | 832 | 0.148187 |
| X3 | -52.6 | 33.47985 | 2058 | 1003 | 0.170412 |
| X30 | -52.5 | 46.38925 | 2058 | 1105 | 5.3592 |
| X31 | -31.4 | 42.78765 | 2058 | 806 | 6.3216 |
| X32 | -37.22 | 48.61934 | 2058 | 1355 | 4.787182 |
| X33 | -41.8 | 38.80279 | 2058 | 1254 | 4.4556 |
| X34 | -40.2 | 62.29344 | 2058 | 1075 | 5.1552 |
| X35 | -42.43 | 41.83244 | 2058 | 1259 | 6.0204 |
| X36 | -48.4 | 38.52776 | 2040 | 2305 | 4.5864 |
| X37 | -30.15 | 44.50015 | 2036 | 1070 | 8.4084 |
| X39 | -37.1 | 32.52637 | 2022 | 878 | 6.9516 |
| X4 | -37.52 | 42.35128 | 2004 | 836 | 7.7208 |
| X5 | -41.6 | 48.87517 | 1980 | 1452 | 6.4752 |
| X57 | -54.43 | 41.59128 | 1890 | 1410 | 0.101066 |
| X6 | -45.52 | 35.94445 | 1842 | 3244 | 0.190489 |
| X60 | -53.25 | 29.62 | 1830 | 898 | 4.092 |
| X71 | -32.4 | 28.04358 | 1698 | 735 | 0.136526 |
| X72 | -51.03 | 42.02831 | 1644 | 878 | 0.158687 |
| X85 | -32.6 | 54.62329 | 1482 | 1253 | 5.1264 |
| X88 | -31.6 | 33.91691 | 1278 | 2509 | 0.1488 |
| X89 | -28.6 | 13.73 | 1224 | 1452 | 3.456 |
| X9 | -11.6 | 37.28185 | 1000 | 7991 | 2.0556 |
